# Cytoplasmic long noncoding RNAs are differentially regulated and translated during human neuronal differentiation

**DOI:** 10.1261/rna.078782.121

**Published:** 2021-09

**Authors:** Katerina Douka, Isabel Birds, Dapeng Wang, Andreas Kosteletos, Sophie Clayton, Abigail Byford, Elton J.R. Vasconcelos, Mary J. O'Connell, Jim Deuchars, Adrian Whitehouse, Julie L. Aspden

**Affiliations:** 1 School of Molecular and Cellular Biology, Faculty of Biological Sciences, University of Leeds, Leeds LS2 9JT, United Kingdom; 2 LeedsOmics, University of Leeds, Leeds LS2 9JT, United Kingdom; 3 School of Biomedical Sciences, Faculty of Biological Sciences, University of Leeds, Leeds LS2 9JT, United Kingdom; 4 Astbury Centre for Structural Molecular Biology, University of Leeds, Leeds LS2 9JT, United Kingdom; 5 School of Life Sciences, Faculty of Medicine and Health Sciences, The University of Nottingham, Nottingham NG7 2UH, United Kingdom

**Keywords:** lncRNA, neuronal differentiation, polysome, ribosome profiling, translation

## Abstract

The expression of long noncoding RNAs is highly enriched in the human nervous system. However, the function of neuronal lncRNAs in the cytoplasm and their potential translation remains poorly understood. Here we performed Poly-Ribo-Seq to understand the interaction of lncRNAs with the translation machinery and the functional consequences during neuronal differentiation of human SH-SY5Y cells. We discovered 237 cytoplasmic lncRNAs up-regulated during early neuronal differentiation, 58%–70% of which are associated with polysome translation complexes. Among these polysome-associated lncRNAs, we find 45 small ORFs to be actively translated, 17 specifically upon differentiation. Fifteen of 45 of the translated lncRNA-smORFs exhibit sequence conservation within *Hominidea*, suggesting they are under strong selective constraint in this clade. The profiling of publicly available data sets revealed that 8/45 of the translated lncRNAs are dynamically expressed during human brain development, and 22/45 are associated with cancers of the central nervous system. One translated lncRNA we discovered is *LINC01116*, which is induced upon differentiation and contains an 87 codon smORF exhibiting increased ribosome profiling signal upon differentiation. The resulting LINC01116 peptide localizes to neurites. Knockdown of *LINC01116* results in a significant reduction of neurite length in differentiated cells, indicating it contributes to neuronal differentiation. Our findings indicate cytoplasmic lncRNAs interact with translation complexes, are a noncanonical source of novel peptides, and contribute to neuronal function and disease. Specifically, we demonstrate a novel functional role for *LINC01116* during human neuronal differentiation.

## INTRODUCTION

Long noncoding RNAs (lncRNAs) are >200 nt in length and thought to lack the ability to encode proteins. LncRNAs are less conserved, yet more tissue- and developmental-stage-specific than mRNAs (Tsagakis et al. 2020). Early work indicated that the majority of lncRNAs were predominantly nuclear and localized to chromatin (Derrien et al. 2012; Djebali et al. 2012). However, it has become increasingly clear that many lncRNAs are exported to the cytoplasm, and recent estimates are that ∼54% of lncRNAs are detected in the cytoplasm (Carlevaro-Fita et al. 2016). Although many lncRNAs have been found to bind proteins, biological functions have only been determined for a relatively small number of lncRNAs.

Several lncRNAs have been found to play key roles in development and differentiation; for example, *lnc-31* during myoblast differentiation (Dimartino et al. 2018). They are particularly enriched in the nervous system of *Drosophila melanogaster*, mouse and human. It is estimated that ∼40% of human lncRNAs are specifically expressed in the brain (Derrien et al. 2012), where they display precise spatiotemporal expression profiles (Ponting et al. 2009). A subset of nuclear neuronal lncRNAs have been found to regulate neuronal differentiation in mouse and human (Chodroff et al. 2010; Lin et al. 2014; Winzi 2018; Carelli et al. 2019). However, only a small number of cytoplasmic lncRNAs have had their biological and molecular functions elucidated. These include lncRNAs found to associate with translation complexes (Carrieri et al. 2012) and to have specific cytoplasmic functions in posttranscriptional gene regulation. For example, *BACE1-AS* transcript, which is significantly up-regulated in the brains of Alzheimer's disease patients, base-pairs with *beta-secretase-1* (*BACE1*) mRNA, stabilizing it (Faghihi et al. 2010), whereas *BC200* represses translation initiation in dendrites by disrupting the formation of preinitiation 48S complexes (Wang et al. 2002). Together these studies indicate that there may be many lncRNAs present in the cytoplasm, potentially playing important roles during neuronal development and differentiation that are yet to be discovered.

Ribosome profiling (Ribo-Seq) in a range of organisms and tissue types has revealed the translation of a variety of noncanonical ORFs, including small ORFs (smORFs) <100 codons in length from within lncRNAs (Guo et al. 2010; Ingolia et al. 2013; Aspden et al. 2014; Duncan and Mata 2014; Blair et al. 2017; Fujii et al. 2017; Rodriguez et al. 2019). Although these translation events remain controversial, it is clear that lncRNAs can interact with the translation machinery (Ruiz-Orera and Alba 2019). Limited ribosome profiling signal found on smORFs might be the result of sporadic binding of a single ribosome and may not necessarily correspond to active translation (Patraquim et al. 2020). We previously developed Poly-Ribo-Seq to distinguish those lncRNAs that are bound by multiple ribosomes, and therefore actively translated, from nonspecific background signal (Aspden et al. 2014). A small but growing number of smORF peptides translated from lncRNAs have been found to exhibit cellular and organismal functions (Pueyo and Couso 2008; Magny et al. 2013; Anderson et al. 2015; Chen et al. 2020; Spencer et al. 2020; Wang et al. 2020). Therefore, the identification of genuine smORF translation events from lncRNAs by ribosome profiling is an important first step in assessing the wider importance of these smORF peptides. To date, a robust assessment of lncRNA translation in the context of neuronal differentiation, where lncRNA expression is enriched, has been missing.

Given (i) the large number of lncRNAs enriched in the human central nervous system, (ii) recently revealed lncRNA roles in differentiation, and (iii) evidence of translation of lncRNA to produce small peptides, we reasoned that lncRNAs may functionally interact with polysomes and be translated during neuronal differentiation. This work aimed to probe the dynamic interactions of lncRNAs with the translation machinery and identify actively translated cytoplasmic lncRNAs during early neuronal differentiation. This will be important to understand the biological role of cytoplasmic lncRNAs and to identify novel peptides with potentially biological and medically important functions.

Here, we have performed Poly-Ribo-Seq (Aspden et al. 2014), an adaptation of ribosome profiling, to determine the population of lncRNAs present in the cytoplasm, assess their interaction with the translation machinery, and establish which lncRNAs are translated. We used the human neuronal cell line SH-SY5Y to provide a model of neuronal differentiation and to generate sufficient material for Poly-Ribo-Seq. We followed up our transcriptome wide analysis probing a subset of candidate lncRNAs in more detail in terms of their enrichment in the cytoplasm, precise association with translation complexes, and ORF tagging experiments. For the translated lncRNAs we identified, we have assessed their conservation and their importance to neuronal development and disease using previously published data sets. For one translated candidate lncRNA, *LINC01116*, we characterized its functional contribution to neuronal differentiation.

## RESULTS

### Differentiation of SH-SY5Y cells with retinoic acid results in reduced translation levels

To dissect the importance of cytoplasmic lncRNAs and their ribosome associations in early neuronal differentiation, we profiled the differentiation of SH-SY5Y cells with retinoic acid (RA) for 3 d. This treatment results in neuronal differentiation as indicated by neurite elongation, which can be seen by immunostaining for neuronal βIII-tubulin (TuJ1) (Supplemental Fig. 1A), and quantification of neurite length reveals significant elongation upon RA treatment (Supplemental Fig. 1B). There is also increased expression of neuronal markers: more cells express c-Fos upon differentiation (Supplemental Fig. 1C,D). There is a concomitant reduction in cell proliferation, as seen by the reduced number of Ki67+ cells (Supplemental Fig. 1E,F). Together this data indicates that our RA treatment of SH-SY5Y cells leads to neuronal differentiation.

To determine if this RA-induced differentiation of the SH-SY5Y model will be suitable to study translational dynamics, the translational output of these cells was assessed by polysome profiles (Supplemental Fig. 2A). This revealed that differentiation results in down-regulation of global translation. Quantification of translation complexes across the sucrose gradients indicates that levels of polysomes are reduced with respect to 80S monosomes, resulting in a reduced polysome to monosome ratio (Supplemental Fig. 2B). This down-regulation of translation is accompanied by a shift of ribosomal protein (RP) mRNAs from polysomes to monosomes: for example, *RPL26* mRNA (Supplemental Fig. 2C), *RPS28* (Supplemental Fig. 2D), and *RPL37* (Supplemental Fig. 2E), as measured by RT-qPCR across gradient fractions. This reduced synthesis of RPs has previously been reported during neuronal differentiation (Blair et al. 2017; Chau et al. 2018). Together these data indicate that the model of RA-induced neuronal differentiation of SH-SY5Y cells, with dynamically regulated translation, provides an ideal system in which to study cytoplasmic RNAs, their interaction with the translation machinery, and contribution to neuronal differentiation.

### Cytoplasmic lncRNA expression is regulated during neuronal differentiation

**FIGURE 1.**
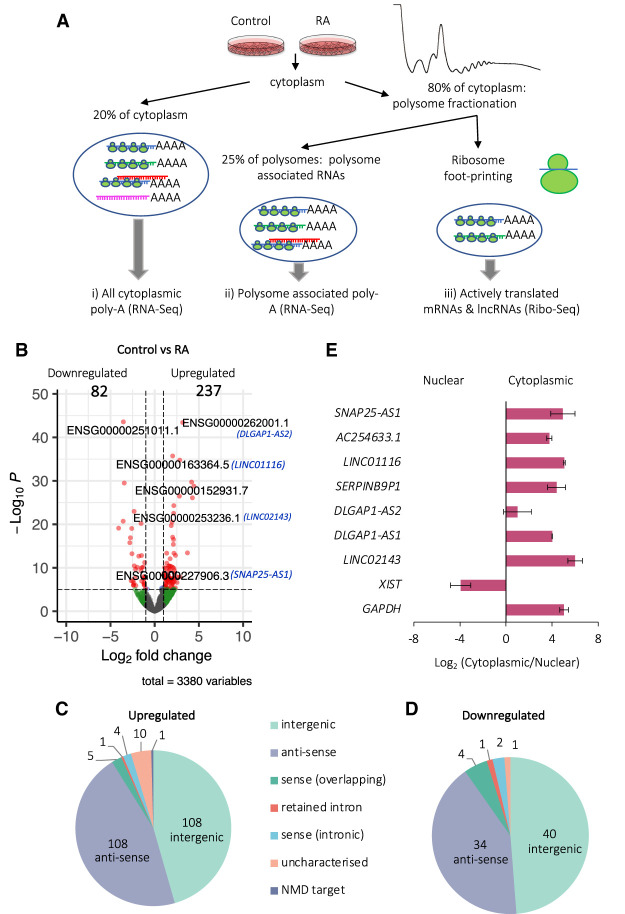
Cytoplasmic lncRNA expression is regulated during neuronal differentiation. (*A*) Schematic of Poly-Ribo-Seq, with three levels of analysis: (i) total cytoplasmic, (ii) polysome-associated, and (iii) translated lncRNAs. (*B*) Volcano plot of differential expression analysis of polysome-associated lncRNAs (labeled by geneIDs and names for candidate lncRNAs) between Control and RA populations. Two hundred and thirty-seven lncRNAs are up-regulated during differentiation and 82 down-regulated (log_2_ fold-change cutoff = 1, *P*^adj^ < 0.05). Pie chart of types of lncRNAs (*C*) up-regulated and (*D*) down-regulated upon differentiation (intergenic; antisense; sense-overlapping; retained intron; sense-intronic; uncharacterized; NMD target). (*E*) LncRNAs of interest that are induced are specifically localized to cytoplasm as shown by subcellular fractionation RT-qPCR. *XIST* lncRNA was used as a nuclear and *GAPDH* mRNA as a cytoplasmic positive control (*n* = 3, SE is plotted, Student's *t*-test, *n* = 3, *P* > 0.05).

To profile RNA, ribosome association, and translational changes upon differentiation, we used Poly-Ribo-Seq (Aspden et al. 2014) with some minor modifications to adapt to human neuronal cells (Fig. 1A). This adaptation of ribosome profiling (Ribo-Seq) can detect which RNAs are (a) cytoplasmic, (b) polysome-associated, and (c) translated (Fig. 1A). We sequenced (i) poly(A) selected cytoplasmic RNA, “Total” RNA-seq, (ii) polysome-associated poly(A) RNAs, “Polysome” RNA-seq, and (iii) ribosomal footprints, Ribo-Seq, from “Control” and “RA” differentiated cells (Fig. 1A).

To determine which lncRNAs are expressed, present in the cytoplasm, and regulated during differentiation, we first analyzed total RNA-seq (Supplemental Fig. 3A). PCA revealed that RA treated samples cluster separately from Control samples and biological replicates generally cluster together (Supplemental Fig. 3B). We detected large numbers of lncRNAs expressed and present (i.e., have RPKMs ≥ 1) in the cytoplasm. In the Control conditions there were 801 lncRNA genes expressed in the cytoplasm and 916 lncRNA genes in differentiated cells. To understand the potential role and regulation of cytoplasmic lncRNAs during neuronal differentiation, we performed differential expression analysis between Control and RA samples at the gene level. We observed 178 lncRNA genes up-regulated and 100 down-regulated during differentiation in the total cytoplasm (Supplemental Fig. 3C). We also performed differential expression analysis at the gene level for Polysomal RNA-seq samples (Fig. 1A). Within the Polysomes, we identified 237 lncRNA genes that were up-regulated during differentiation while only 82 were down-regulated (Fig. 1B). Comparing the lncRNAs differentially regulated in Total and Polysomes populations, the majority, i.e., 71% of the up-regulated (126/178) and 71% of the down-regulated lncRNAs (58/82) found in Polysomes were also found in Total (Supplemental Fig. 3D,E). Significant induction of specific lncRNAs during differentiation, such as *DLGAP1-AS2*, is suggestive of a regulatory role during neuronal differentiation (Supplemental Fig. 3F). An assessment of the types of lncRNA regulated during neuronal differentiation revealed that the majority are either intergenic or antisense lncRNAs, 216/237 for up-regulated (Fig. 1C) and 74/82 for down-regulated (Fig. 1D). In summary, neuronal differentiation results in differential expression of ∼300 lncRNAs within the cytoplasm.

We validated the differentiation-induced changes in a subset of seven lncRNAs (Supplemental Fig. 4A). By RT-qPCR the expressions of 6/7 candidate lncRNAs were significantly (*P* < 0.05: *SNAP25-AS1*, *LINC001116*; *P* < 0.01: *ACE254633.1*, *DLGAP1-AS2*, *DLGAP1-AS1*, *LINC02143*) up-regulated upon differentiation, as was determined by RNA-seq analysis. Fold-changes were highly correlative between RNA-seq and RT-qPCR (Supplemental Fig. 4B). To enable us to focus on lncRNAs with potential neuronal functions, we selected candidate lncRNAs that exhibited the highest fold increase in levels upon differentiation. To understand whether our candidate lncRNAs are genuine cytoplasmic lncRNAs or whether their cytoplasmic population represents a small minority, we assessed their subcellular distribution. The majority of these candidate lncRNAs (6/7) are specifically enriched in the cytoplasm, rather than the nucleus (Fig. 1E; Supplemental Fig. 4C), in contrast to the known nuclear lncRNA *XIST*. Together these data indicate that ∼900 lncRNAs are localized to the cytoplasm; the majority of these are likely enriched in the cytoplasm and ∼30% of these cytoplasmic lncRNAs are dynamically expressed during neuronal differentiation.

### Association of lncRNAs with polysomes during neuronal differentiation

**FIGURE 2.**
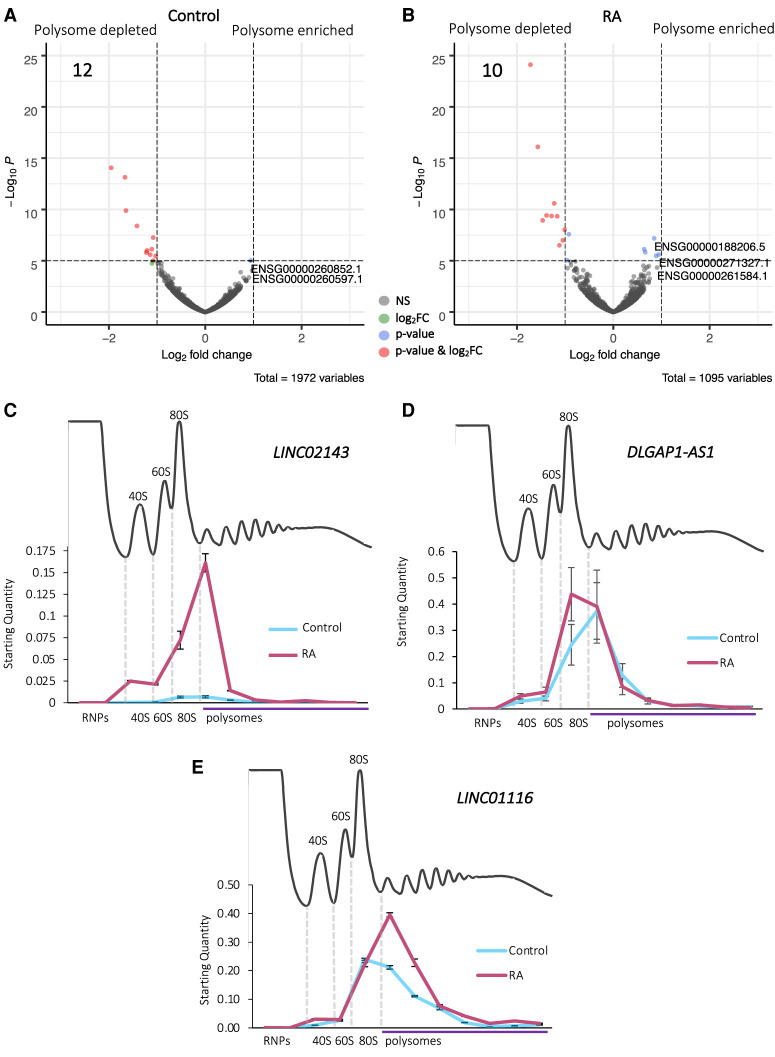
Association of lncRNAs polysomes during neuronal differentiation. Volcano plots displaying the significantly differentially localized lncRNAs (labeled by their geneIDs) between Total and Polysome populations in (*A*) Control and (*B*) RA, with log_2_ fold-change cutoff = 1, *P*^adj^ < 0.05. (*C*–*E*) RT-qPCR of lncRNAs across sucrose gradient fractions (*n* = 3, SE is plotted). (*C*) *LINC02143* is found in 80S and small polysome fractions during differentiation. Five percent of the transcript is detected in 80S (monosome) fractions and 66% in small polysome complexes. (*D*) *DLGAP1-AS2* is found in 80S and small polysome fractions both in control and RA treated cells. On average, 63% of the transcript is detected in the polysome fractions in Control and 49% upon differentiation. (*E*) *LINC01116* is found in 80S and two to seven polysome fractions both in control and RA treated cells. On average, 66% of the *LINC01116* transcript is detected in the polysome fractions in Control and 57% upon differentiation.

To determine the propensity of cytoplasmic lncRNAs to associate with translation complexes and how this is regulated during differentiation, we compared lncRNAs levels between the whole cytoplasm (Total-RNA-seq) and the polysomes (Polysome-RNA-seq). This analysis indicated that the vast majority of lncRNAs (Control: 98% and RA: 99%) are neither polysome enriched nor depleted (Fig. 2A,B). This suggests that most lncRNAs are not actively targeted to polysomes but present in translation complexes. A small number (Control: 12 and RA: 10) of lncRNAs are specifically depleted from the polysomes (Fig. 2A,B). This suggests that the roles these lncRNAs play are likely elsewhere in the cytoplasm and not directly connected to translation. Nine of 12 depleted in Control are not depleted upon differentiation, indicating their polysome association is regulated during differentiation (Supplemental Fig. 5A). There is a smaller proportion of antisense lncRNAs within these polysome-depleted populations (Supplemental Fig. 5B,C) as compared to the proportion displayed by those lncRNAs differentially expressed during differentiation (Fig. 1C,D). This may indicate that antisense lncRNAs are more likely to be present in polysomes, and their role in the polysomes could be linked to their antisense characteristics.

To understand the precise nature of the association of our candidate lncRNAs with translation complexes, their distribution was profiled across sucrose gradient fractions. This confirmed that these lncRNAs are found to associate with polysome complexes within the cytoplasm but also reveals the precise translation complexes they interact with in terms of ribosomal subunits, 80S monosomes, and different polysomes. We first profiled *LINC02143*, which is induced >22-fold during differentiation (Supplemental Fig. 4A) and highly enriched in the cytoplasm compared to the nucleus (Fig. 1E). *LINC02143* was mainly found in monosomes (80S) and small polysomes (two to three ribosomes) (Fig. 2C). *DLGAP1-AS1* was also found to associate with small polysomes (two to four ribosomes), as well as ribosomal subunits and 80S monosomes (Fig. 2D). Therefore, both *LINC02143* and *DLGAP1-AS1* could either be translated or regulate translation.

Another lncRNA whose levels significantly increase during differentiation is *LINC01116* (Fig. 1B; Supplemental Fig. 4A), which has previously been shown to be involved in the progression of glioblastoma (GBM) (Brodie et al. 2017). *LINC01116* is enriched in the cytoplasm (Fig. 1E), detected at high levels in the 80S (monosome) fraction and in small and medium polysomes (two to seven ribosomes) (Fig. 2E). Upon differentiation there is an increase in the amount of *LINC01116* present in disomes, compared to Control. This is consistent with the up-regulation of the *LINC01116* transcript in the polysomes detected by RNA-seq, indicating a likely functional interaction of *LINC01116* with polysomes during differentiation. In fact, the majority of the *LINC01116* transcript was found to associate with polysomes in both undifferentiated cells (Control-66%) and upon differentiation (RA-57%), suggesting it is could either be translated or associating with translation complexes (Fig. 2E). Overall, these data indicate that the majority of cytoplasmic lncRNAs are polysome-associated but not enriched. Comparing the differentiation-induced lncRNA changes between Total and Polysome populations, as well as specific candidate lncRNAs, also suggests that polysome association is dynamic during differentiation.

### Translation of lncRNA-smORFs during differentiation

**FIGURE 3.**
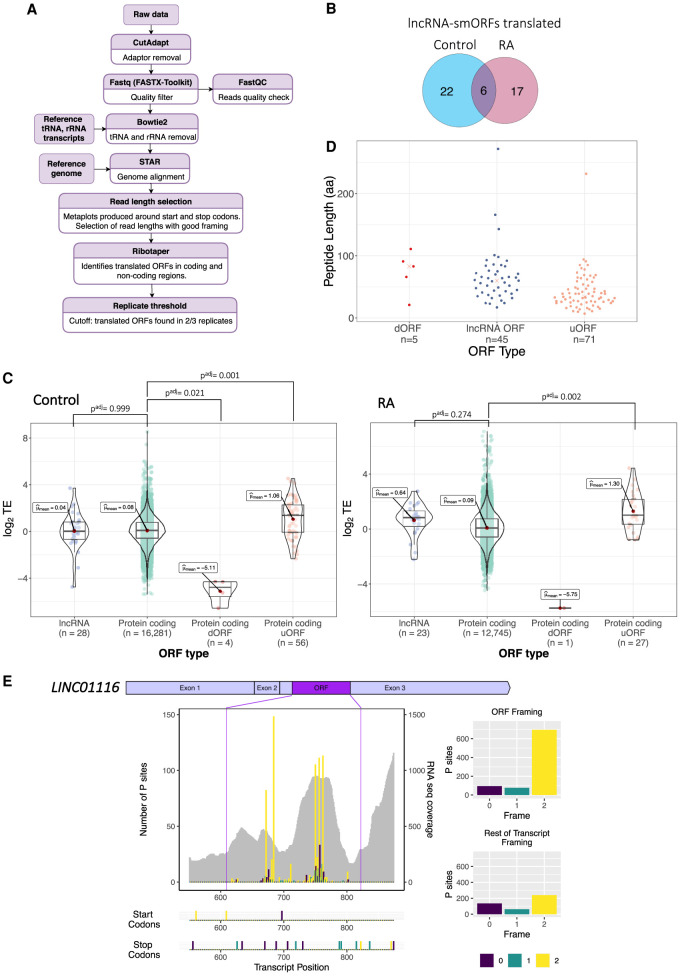
Translation of lncRNA-smORFs. (*A*) Workflow for identification of translated ORFs from Ribo-Seq and RNA-seq; see Materials and Methods for details. (*B*) Venn diagram of lncRNA-smORFs translated in Control and RA, with overlap. (*C*) Plots of translational efficiencies for protein-coding ORFs, lncRNA-smORFs, dORFS, and uORFs. (*D*) Length distribution of translated ORFs in lncRNAs, dORFs, and uORFs (in codons). (*E*) Poly-Ribo-Seq profile for *LINC01116* in RA treatment. RNA-seq (Polysome) reads are gray and ribosome P sites are in purple, turquoise, and yellow according to frame. Purple lines mark beginning and end of translated smORF. All possible start and stop codons are indicated *below*. Framing within and outside translated smORF shown on *left*.

To better understand the association of lncRNAs with polysome complexes and their potential translation, we analyzed our third and final data set, ribosome footprinting from our Poly-Ribo-Seq experiments (Fig. 1A, actively translated mRNAs and lncRNAs). Triplet periodicity analysis reveals good framing, specifically at footprint lengths of 31 and 33 nt (Supplemental Fig. 6A; Supplemental Table 1). On average, 95% of ribosome footprints mapped to CDSs, while in RNA-seq this was only 53% (Supplemental Fig. 6B). Together, these attributes indicate that our Ribo-Seq quality is high and represents genuine translation events. Since 31 and 33 nt reads exhibited high triplet periodicity, they were selected for downstream analysis of translation events to identify ORFs that were translated (Fig. 3A). Overall, we are able to detect the translation of both canonical protein-coding ORFs and noncanonical ORFs, including upstream (uORFs) and downstream ORFs (dORFs), in both Control and RA conditions (Table 1).

**TABLE 1.**
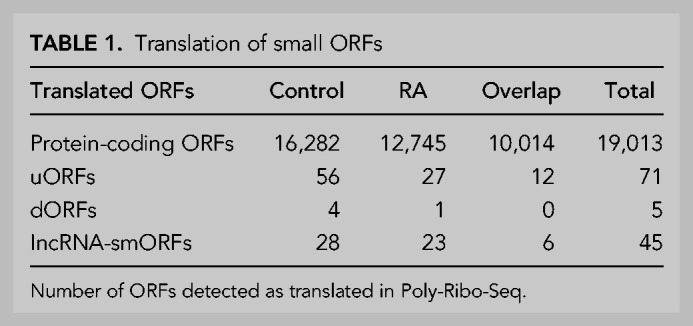
Translation of small ORFs

We analyzed our Ribo-Seq data in order to determine if any of the polysome-associated lncRNAs are engaged with ribosomes and translated. Using our pipeline (Fig. 3A), we detected the translation of 45 small ORFs within lncRNAs (lncRNA-smORFs), 28 in Control and 23 during differentiation; six of these were translated in both conditions (Fig. 3B). Only two of these lncRNA-smORFs have previously been characterized, *CRNDEP* (Szafron et al. 2015) and *HAND2-AS1* (van Heesch et al. 2019); the remaining 43 represent novel ORFs. The level at which these lncRNA-smORFs are translated was assessed by determining their translational efficiencies (TEs), the number of ribosome footprints relative to RNA abundance. The TEs of translated lncRNA-smORFs were similar to those measured for protein-coding ORFs (Fig. 3C), providing further evidence that these are genuine translation events, whereas the dORFs that we detect are translated at much lower efficiencies (Fig. 3C). This is likely to be because ribosomes would have to reinitiate after the main ORF, which would occur at a lower efficiency. As an additional assessment of whether the translation of lncRNA-smORFs represent genuine translation events, we compared the pattern of ribosome footprints across the smORFs with protein-coding CDSs (Supplemental Fig. 6C–F). In both Control and RA conditions, metagene plots show that the distribution of footprints is very similar between lncRNA-smORFs (Supplemental Fig. 6C,D) and protein-coding CDSs (Supplemental Fig. 6E,F), specifically around start and stop codons. There is a substantial drop-off of footprints at the stop codon for both, indicative of genuine translation events. Together, our Ribo-Seq analysis reveals that a subset of polysome-associated lncRNAs is translated.

To better understand these translated lncRNA-smORFs, we profiled their features. Analysis of the 45 translated smORFs from lncRNAs shows that they are all <300 aa in length with a median size of 60 aa (Fig. 3D) (56 aa in Control and 64 aa in RA: Supplemental Fig. 7A). Previous analysis has indicated that *Drosophila* smORF peptides exhibit specific amino acid usage, indicating that they are genuine proteins and show a propensity to form transmembrane α-helices (Aspden et al. 2014). Therefore, we profiled the amino acid composition of our lncRNAs-smORFs, uORFs, and dORFs compared to protein-coding ORFs and expected by chance frequencies (Supplemental Fig. 7B). lncRNA-smORFs exhibit similar frequencies to known protein-coding ORFs. Specifically, smORFs possess lower than expected arginine levels, but not as low as known protein-coding ORFs. Amino acid usage does not suggest that any smORF groups have a propensity to form transmembrane α-helices.

From within the set of lncRNAs we identified as induced during differentiation (RNA-seq), several contained translated smORFs (Ribo-Seq). One of these is *LINC01116*, which contains a 71-codon smORF detected as translated by our Ribo-Seq data. The ribosome profiling signal is substantially higher upon differentiation (Fig. 3E; Supplemental Fig. 7C), mainly as a result of increased lncRNA transcript abundance. Overall, ∼80% of reads that map to this ORF are in frame 2, whereas outside this ORF, the few reads mapping to the remaining lncRNA sequence are far more equally distributed between the three possible frames (Fig. 3E). Such robust framing is highly indicative of genuine translation of this specific smORF, from within the *LINC01116* lncRNA. Together, analysis of our Ribo-Seq data has led to the discovery of 43 novel lncRNA-smORFs with robust indicators of translation.

### Peptide synthesis from smORFs in lncRNAs during differentiation

**FIGURE 4.**
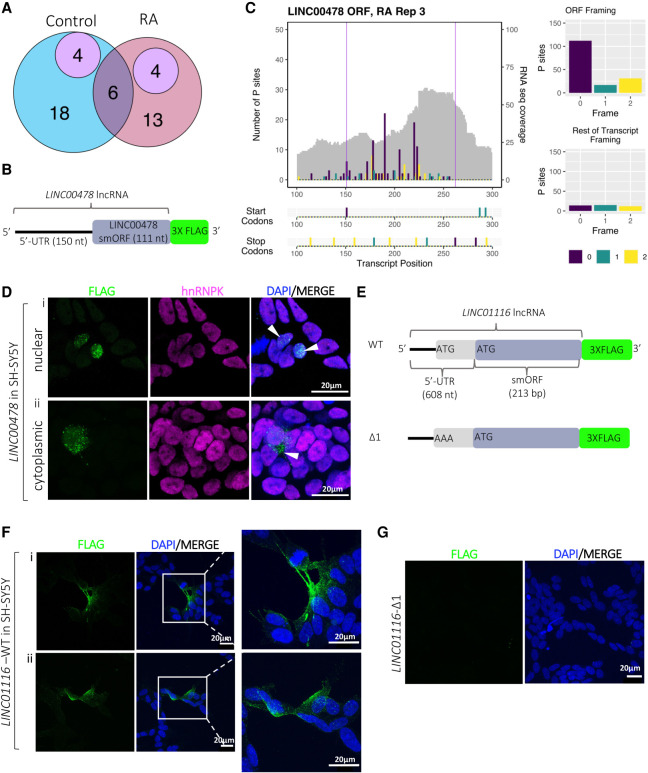
Peptide production from smORFs in lncRNAs. (*A*) Venn diagram showing overlap lncRNA-smORFs detected between our Poly-Ribo-Seq and publicly available mass spectrometry data from SH-SY5Y (purple). Control in blue, RA in pink. (*B*) Schematic of tagging construct for *LINC00478*; lncRNA sequence upstream of smORF and smORF, excluding its stop codon, cloned upstream of 3× FLAG, which is lacking its own start codon. FLAG signal is therefore dependent on smORF translation. (*C*) Poly-Ribo-Seq profile for *LINC00478* in RA treatment. RNA-seq (Polysome) reads are gray and ribosome P sites are in purple, turquoise, and yellow according to frame. Purple lines mark beginning and end of translated smORF. All possible start and stop codons are indicated *below*. Framing within and outside translated smORF shown on *right*. (*D*) Confocal images of FLAG-tagged LINC00478 peptide in SH-SY5Y cells (Control), showing (i) nuclear and (ii) cytoplasmic distribution, green is FLAG, magenta is hnRNPK (marking nuclei), and blue is DAPI (scale bar is 20 µm). (*E*) Schematic of tagging constructs for *LINC01116* (WT and start codon mutant Δ1). (*F*) Confocal images of FLAG-tagged WT LINC01116 peptide showing cytoplasmic localization, near cell membrane and neuritic processes (magnification of insert is 3×). (*G*) Δ1 start codon mutant, showing no FLAG signal, in SH-SY5Y cells; green is FLAG and blue is DAPI (scale bar is 20 µm).

Our pipeline is highly stringent, that is, there are many additional ORFs that display good framing but do not pass our thresholds for numbers of footprinting reads or exhibit background signals in the rest of the lncRNA. Therefore, we are confident these translation events are taking place. To validate peptide synthesis from these translation events, we have taken two complementary strategies: mass spectrometry analysis and transfection of ORF tagging constructs. Analysis of previously published mass spectrometry data sets from SH-SY5Y cells (undifferentiated and RA-treated) (Murillo et al. 2018; Brenig et al. 2020) supports the production of peptides from eight lncRNA-smORFs (four Control and four RA) (Fig. 4A). This relatively low level of support is to be expected, given the small size of these peptides and therefore the reduced chance of producing peptides >8aa from digestion for mass spectrometry detection (Saghatelian and Couso 2015). Overall mass spectrometry supports the peptide synthesis from 18% of lncRNA-smORFs detected by Poly-Ribo-Seq.

To validate translation of our lncRNA-smORFs that were not identified in previous mass spectrometry but were detected as translated by our Poly-Ribo-Seq analyses, we used a transfection tagging approach. We cloned the lncRNA sequence 5′ of the putative ORF, termed the 5′-UTR, and the smORFs, without its stop codon, with a carboxy-terminal 3× FLAG tag (Fig. 4B). The FLAG tag did not have its own start codon, so any FLAG signal is the result of translation from the lncRNA-smORF. Two candidate lncRNA-smORFs were selected that did not have mass spectrometry support: LINC000478 and LINC01116. A 37 codon smORF was detected as translated by our Poly-Ribo-Seq from *LINC00478* in both conditions (Fig. 4C). Transfection of LINC00478-smORF-FLAG into undifferentiated SH-SY5Y cells produced FLAG signal in both nuclear and cytoplasmic compartments (Fig. 4D). FLAG signal was also seen when LINC00478-smORF-FLAG transfected SH-SY5Y cells were treated with RA (Supplemental Fig. 7A). This RA FLAG signal was only ever detected in the nucleus. Similar results were seen in HEK293 cells (Supplemental Fig. 7B), but because of the higher transfection efficiency in HEK293 compared with SH-SY5Y cells, we detected FLAG signal in more cells. Together this indicates that translation of LINC00478-smORF results in the synthesis of peptide, and the specific localization of this peptide is indicative of peptide function.

The second candidate lncRNA-smORF detected by our Poly-Ribo-Seq that we tagged was in *LINC01116*. Tagging of this LINC01116-smORF (Fig. 4E) generated a FLAG signal in the cytoplasm of SH-SY5Y cells, which is localized to neurites (Fig. 4F). A FLAG signal was also present in *LINC01116* transfections in HEK293 cells (Supplemental Fig. 7C), but because of the higher transfection efficiency in HEK293 compared with SH-SY5Y cells, we detected a FLAG signal in more cells.

The LINC01116-smORF detected by Poly-Ribo-Seq is 71 codons in length, but inspection of the lncRNA sequence upstream of the smORF reveals a second potential ATG start codon (Fig. 4E; Supplemental Fig. 7D). Although there was little Ribo-Seq signal to support this 5′ start codon, it is possible that translation of LINC01116-smORF initiates there. The two potential start codons were assessed for similarity to the Kozak sequence consensus, using NetStart1.0 (Pedersen and Nielsen 1997); both exhibited scores >0.5, indicating that both are in good context and therefore either could be used to initiate translation (AUG_1_ = 0.545, AUG_2_ = 0.645). Given the scanning model of translation initiation it seems likely that the first AUG would be used. To determine if the 5′ start codon was used, it was mutated and the effect on production of a FLAG signal measured (Fig. 4E). No FLAG signal was present in transfections where the 5′ start codon was mutated (Δ1) (Fig. 4G). This suggests that the first start codon is necessary for the translation of the LINC01116-smORF and the resulting peptide is 87aa long. Although the FLAG signal is present in a low number of cells, no transfection controls and Δ1 indicate that the FLAG signal is dependent on translation of the LINC01116-smORF (Supplemental Fig. 7E). These results indicate that translation of LINC01116-smORF results in peptide synthesis and this 87aa peptide exhibits a distribution suggestive of a function in neuronal differentiation.

### Translated lncRNA-smORFs exhibit sequence conservation

Another indicator of coding potential and of peptide functionality is sequence conservation. Therefore, we assessed the extent to which the sequences of our novel lncRNA-smORFs are conserved. Given that lncRNAs in general are poorly conserved, we used closely related species to humans: the other four great apes (*P. abelii, P. paniscus, P. troglodytes, G. gorilla*) as well as *N. leucogenys* (ape) and *M. musculus*. To ensure detection of sequence conservation for these short smORFs irrespective of annotation in other genomes, we used three complementary BLAST strategies using the transcript nt sequence, smORF nt sequence, and protein aa sequence.

**FIGURE 5.**
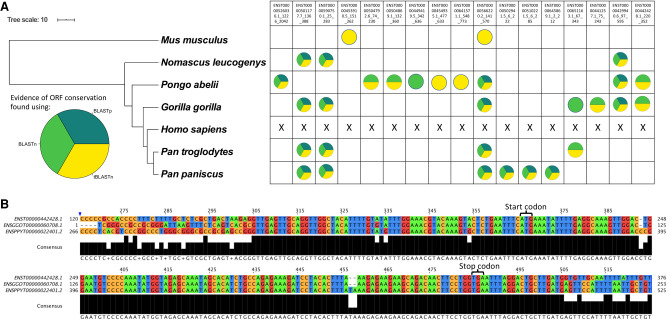
Translated lncRNA-smORFs exhibit sequence conservation in great apes. (*A*) Phylogram with lncRNA-smORFs for which evidence of sequence conservation was found represented as circles, colored according to how sequence conservation was identified. Phylogram built in iTOL (Letunic and Bork 2011) using data from TimeTree (Kumar et al. 2017), scale in 10 MYA. (*B*) Portion of human *ENST00000442428* lncRNA nt alignment with gorilla and orangutan nt sequences, showing the smORF. Alignment built in ClustalOmega (Sievers et al. 2011) displayed in JalView (Waterhouse et al. 2009).

Initial searches using the entire lncRNA transcript sequence (nt) and BLASTn (Altschul et al. 1990) returned results for ∼78% of translated lncRNAs (35/45), many of which had short alignment lengths of 30–100 nt. Although some of these results may represent conservation of the smORFs, many are due to small areas of sequence overlap along the rest of the lncRNA. LncRNAs rarely exhibit the same levels of conservation as mRNAs (Johnsson et al. 2014) but may contain short “modules” of higher sequence conservation, as described for *XIST* lncRNA (Brockdorff 2018). To take account of this, a second round of searches was performed on the initial search results, using the nt sequence of the smORF (BLASTn) (Altschul et al. 1990) followed by manual cross validation. This identified 14 lncRNA-smORFs as exhibiting nt sequence conservation in at least one of the apes or mouse (Fig. 5A). For the majority of these conserved lncRNA-smORFs, conservation is high across the smORF sequence and lower across the rest of the transcript. One such example is AL162386.2-smORF (*ENST00000442428.1*); it exhibits high sequence conservation when compared to gorilla (*G. gorilla*) and orangutan (*P. abelii*), with 100% and 99% smORF nt sequence identity, respectively (Fig. 5B). When entire transcripts are aligned, this percentage sequence identity drops to 74% with gorilla (*ENSGGOT00000060708.1*), and 65% with orangutan (*ENSPPYT00000022401.2*), indicating the smORF is the most conserved part of these transcripts. Together this suggests that the AL162386.2-smORF, like canonical protein-coding CDSs is under greater selective pressure than its UTRs.

To further corroborate these results, a tBLASTn (Altschul et al. 1990) search of the lncRNA-smORF aa sequences was performed, which uses translated transcript databases in all six frames. This removes the noise of synonymous substitutions, which can have a significant effect, particularly in smORFs (Ladoukakis et al. 2011). For the majority of smORFs, the same results were returned as the first BLASTn strategy, and evidence of conservation was found for a further three lncRNA-smORFs (ENST00000454935.1_477_633, ENST00000557660.5_42_186, ENST00000453910.5_151_262) that appear to have undergone some frameshift mutations (Fig. 5A).

The third BLAST strategy at the aa sequence level, using BLASTp (Altschul et al. 1990), identified homologous peptide sequences for 18% (8/45) of the translated lncRNA-smORFs. This strategy only identifies peptide sequences previously annotated at protein-coding regions in the search species. However, all the returned proteins were unreviewed, uncharacterized proteins, with no evidence at protein, transcript, or homology levels in the Uniprot database (Bateman et al. 2019). This potentially suggests that automated annotation pipelines recognized the coding potential of these smORFs, unlike in the more curated human genome annotation. Overall, the combination of these three layers of sequence conservation analysis, as assessed by three BLAST strategies, revealed that 12 of our translated lncRNA-smORFs exhibit sequence conservation within *Hominidae*, three additional smORFs are also found in gibbons (Fig. 5B), with evidence for two translated smORFs detectable at the greater evolutionary distance of human to mouse, but not found in all apes (Fig. 5A). In total, we have discovered homologs for 17/45 of the lncRNA-smORFs in at least one of the searched species (Fig. 5A). Together this analysis indicates 38% of translated lncRNA-smORFs exhibit some level of sequence conservation, indicating that it is likely that they are translated in other species.

We took advantage of the identification of homologous smORFs in other species to determine if our translated smORFs exhibit signals of conserved protein-coding regions. Multispecies nt sequence alignments were made and phylogenetic codon analysis performed with PhyloCSF (Lin et al. 2011). Of the 17 lncRNA-smORFs with homologs in other species, 14 could be assessed, based on the organisms that the homologs were identified in. Four smORFs exhibited positive PhyloCSF scores, indicating that they are likely to represent a conserved coding region, while the other 10 had negative scores (Supplemental Table 2). The four positive scoring lncRNA-smORFs are in *AC020928.2*, *ENTPD1-AS1*, *LINC00839*, and *THAP9-AS1*. This analysis provides further support that these four lncRNA-smORFs likely encode peptides.

### Translated lncRNAs are associated with neuronal functions and diseases

**FIGURE 6.**
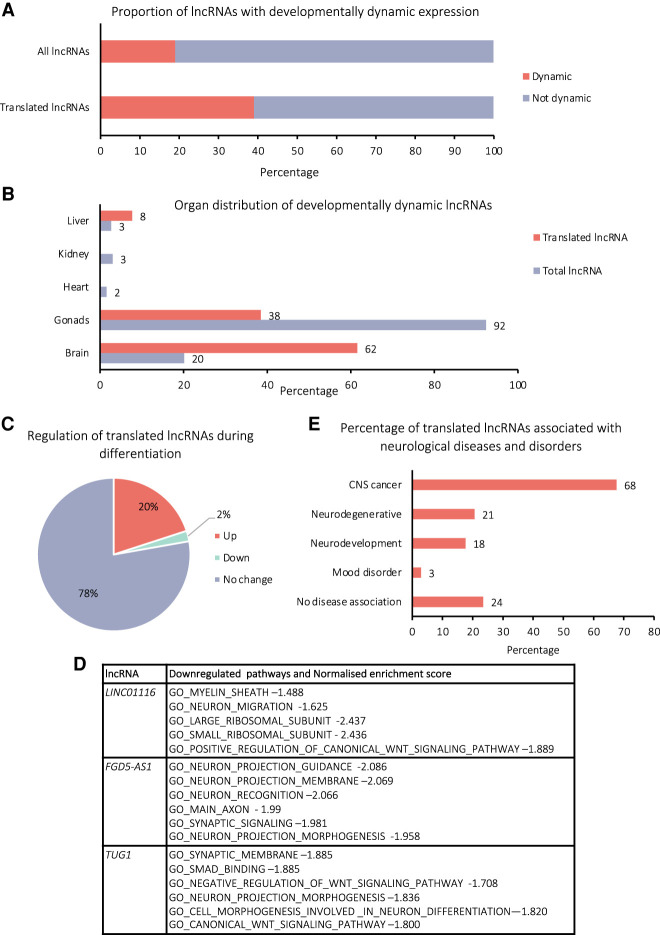
Potential biological importance of translated lncRNAs in neural development, differentiation, and disease. (*A*) Percentage of translated lncRNA genes and all lncRNA genes, which exhibit dynamic expression during human development according to lncExpDB (Sarropoulos et al. 2019). (*B*) Percentage of the dynamically expressed, translated lncRNAs, which show dynamic expression in each organ, for total and translated lncRNA populations, according to LncExpDB (Sarropoulos et al. 2019). (*C*) Proportion of translated lncRNAs, which exhibit differential expression during differentiation of SH-SY5Y cells. (*D*) GO terms associated with changes in RNA-seq levels upon siRNA knockdown of three of the translated lncRNAs (*LINC01116*, *FGD5-AS1*, and *TUG1*) performed by FANTOM6 in human dermal fibroblasts. (*E*) Percentage of translated lncRNA genes found to be associated with neuronal diseases and disorders according to lncRNADisease, Differential Expression Atlas, Cancer RNA-Seq Nexus, Malacards.

To probe the potential biological function, expression, and association with human disease, we profiled the 45 translated lncRNAs using published data. Dynamic expression of lncRNAs can indicate tissues and developmental time points when lncRNAs may function. Therefore, we first assessed whether our translated lncRNA genes exhibited developmentally dynamic expression. In lncExpDB (Sarropoulos et al. 2019), genes that show large changes in expression at different time points during development of a specific organ are annotated as “dynamic.” Thirty-nine percent of our translated lncRNA genes exhibit “dynamic” expression compared with 19% of the total population of lncRNA genes present in lncExpDB (Fig. 6A). This indicates that translated lncRNA genes are more likely to have developmentally regulated expression than lncRNAs in general, suggesting biological roles for these lncRNAs.

We found that 62% of these dynamic translated lncRNAs are regulated in the brain, compared with 20% of all dynamic lncRNAs (Fig. 6B), therefore our translated lncRNAs may function in the brain during development. When we consider our own RNA-seq of Control and RA treated SH-SY5Y cells, 22% of the translated lncRNAs exhibit differential expression in the cytoplasm, 20% up-regulated and 2% down-regulated upon differentiation (Fig. 6C). This potentially indicates that the biological role of these translated lncRNAs may be of broader neuronal importance than just in this differentiation model.

By examining data from the FANTOM6 project, we were able to probe potential cellular functions for three of our translated lncRNAs. siRNA knockdowns of *LINC01116*, *FGD5-AS1*, and *TUG1* were performed in human dermal fibroblasts followed by RNA-seq to understand global effects of depleting these lncRNAs. GO term analysis of these published data showed that genes associated with neuronal function were enriched for all three of our translated lncRNAs (Fig. 6D). This is particularly striking given knockdown was performed in a nonneuronal cell type and suggests all three lncRNAs possess neuronal functions.

To investigate potential roles for our translated lncRNAs in human disease, we examined published association studies specifically for neurological diseases and disorders (Chen et al. 2013; Li et al. 2016; Rappaport et al. 2017; Bao et al. 2019). This revealed 68% of the translated lncRNAs have an association with cancers of the central nervous system (CNS) (Fig. 6E). This is consistent with our discovery of their translation in a neuroblastoma cell line (SH-SY5Y). In addition, 21% of the translated lncRNAs are associated with neurodegenerative diseases and 18% with neurodevelopmental disorders (Fig. 6E). Overall examination of published data on our translated lncRNA indicates that they likely have neuronal functions, potentially during neuronal development, and may contribute to neuronal diseases.

### *LINC01116* contributes to neuronal differentiation

**FIGURE 7.**
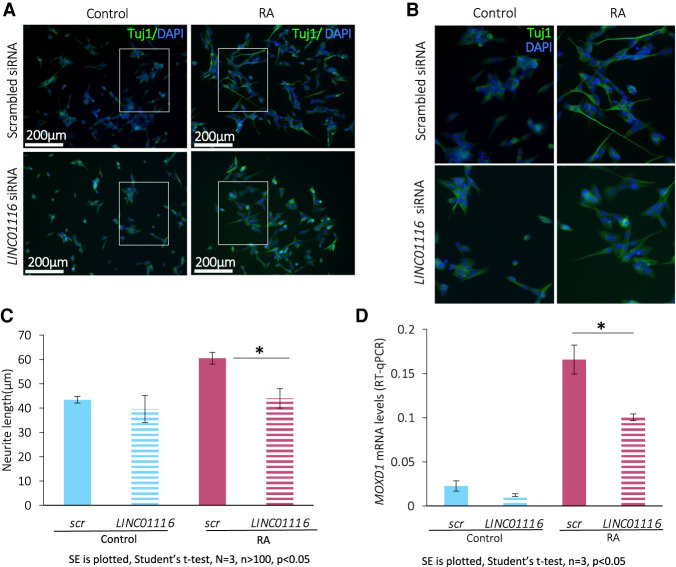
*LINC01116* contributes to neuronal differentiation but does not affect cell cycle progression. (*A*) Representative immunofluorescence images of Control and RA SH-SY5Y cells, transfected with siRNA targeting *LINC01116* and scrambled control, after staining for Tuj (βIII-tubulin) at day 3 post-differentiation (scale bar = 200 µm). White windows magnified in *B*. (*C*) Quantification of neurite length in Control and RA treated cells upon knockdown shows a significant reduction of neurite length in the differentiated cells upon *LINC01116* knockdown (*N* = 3 biological replicates, *n* > 100 measurements, Student's *t*-test *P* < 0.05). (*D*) RT-qPCR of differentiation marker *MOXD1* in Control and RA treated cells, transfected with siRNA targeting *LINC01116* and scrambled control, shows significant reduction of *MOXD1* expression in differentiated cells with reduced *LINC01116* levels at day 3 post-differentiation (*n* = 3 biological replicates, Student's *t*-test *P* < 0.05).

To dissect the potential role of the translated lncRNAs during neuronal differentiation, we performed siRNA knockdown in SH-SY5Y cells. We selected the candidate lncRNA *LINC01116* because we discovered it is induced during differentiation and translated to produce a neurite localized peptide. We performed *LINC01116* siRNA knockdown in both undifferentiated and differentiated SH-SY5Y cells, achieving an 89%–94% reduction in *LINC01116* levels (Supplemental Fig. 8A). *LINC01116* knockdown had a limited effect on cell viability (Supplemental Fig. 8B). The extent of differentiation was then assessed by Tuj1 immunofluoresence, which revealed that *LINC01116* knockdown resulted in a significant reduction of neurite length in RA treated SH-SY5Y cells (Fig. 7A, zoom in Fig. 7B), compared to scrambled siRNA treated SH-SY5Y (Fig. 7C). However, there was no effect of the knockdown in undifferentiated cells (Fig. 7C). This suggests that *LINC01116* is involved in the regulation of neuritic processes formation during neuronal differentiation. To examine potential effects of *LINC01116* knockdown further on differentiation, we assessed the expression levels of the noradrenergic marker *MOXD1*, which is important in neural crest development. *LINC01116* siRNA knockdown, upon differentiation, resulted in a reduction of *MOXD1* expression levels, further indicating a role of *LINC01116* in neuronal differentiation (Fig. 7D). However, *LINC01116* knockdown had no effect on proliferation, as measured by percentage of Ki67+ cells (Supplemental Fig. 8C,D) or cell cycle, as measured by *E2F1* mRNA RT-qPCR (Supplemental Fig. 8E). *LINC01116* likely functions early in the differentiation pathway since its levels are significantly up-regulated within the first 24 h of RA-induced differentiation (Supplemental Fig. 8F). Expression of *LINC01116* then declines rapidly by day 8 (Supplemental Fig. 8G). Together these results suggest that *LINC01116* functions during early differentiation, contributing to neurite process formation.

## DISCUSSION

In this work, we have dissected the relationship of lncRNAs with the translation machinery during human neuronal differentiation using RA treated SH-SY5Y cells as a model. We discovered that ∼800–900 lncRNA genes are expressed and exported to the cytoplasm. A total of 85%–90% of these cytoplasmic lncRNAs are associated with polysome complexes, suggesting that they are either being translated or regulating the translation of the mRNAs with which they interact. Moreover, the association of lncRNAs with polysomes is dynamic during differentiation, as shown by the differential polysome enrichment of lncRNAs in Control and RA treated cells. These results reveal that many lncRNAs are present in the cytoplasm, enriched there, and associated with translation complexes.

We characterized *LINC02143* in more detail, which was found to associate with polysomes. It is an intergenic lncRNA with no known function, which is induced upon differentiation. It is detected in 80S and small polysome fractions, indicating it interacts with the translation machinery, but it is not detected as translated. A number of antisense polysome-associated lncRNAs appear to be up-regulated upon differentiation. Among them is *DLGAP1-AS1*, which is antisense to *Disks large-associated protein 1* (*DLGAP1*), a protein-coding gene involved in chemical synaptic transmission. *DLGAP1-AS1* interacts with actively translating polysomes both in Control and upon differentiation, but it is not translated. The lncRNAs depleted from the polysomes have fewer antisense lncRNAs relative to other populations, suggesting that antisense lncRNAs are preferentially localized to polysomes. These polysome-associated antisense lncRNAs could potentially regulate the translation of their “sense” mRNA through base-pairing, as is the case with *BACE1-AS* (Faghihi et al. 2010) and *UCHL1-AS* (Carrieri et al. 2012).

Ribosome profiling of the actively translating polysomes allowed us to distinguish between the lncRNAs that simply associate with the polysome complexes and those that are being actively translated. We identified 45 translated lncRNA-smORFs, 43 of which are novel ORFs. These translated lncRNA-smORFs exhibit high levels of triplet periodicity, and their translational efficiencies are similar to protein-coding genes. We can therefore be confident that these are real translation events leading to the production of substantial peptide levels rather than background, spurious events (Guttman et al. 2013; Bazzini et al. 2014; Ruiz-Orera and Alba 2019; Patraquim et al. 2020). The size distribution of our novel translated ORFs indicates that the majority are indeed smORFs (<100aa). The general pattern we identified is that dORFs > lncRNA-smORF > uORFs in size. This is consistent with previous studies where a wide range of peptide lengths were discovered (Aspden et al. 2014; Chong et al. 2020). Amino acid composition of these translated smORFs supports the fact they are translated into peptides. However, it does not suggest they are enriched for transmembrane α-helices, in contrast to the smORFs characterized in *D. melanogaster* (Aspden et al. 2014).

Overall, we have independent evidence for peptide synthesis for 12/45 lncRNA-smORFs. Eight of these are from published mass spectrometry data from SH-SY5Y cells (Brenig et al. 2020). In general, we find our lncRNA-smORFs translated in the same treatment (undifferentiated or differentiated) as these mass spectrometry data sets detect the smORF peptides (7/8). An 18% mass spectrometry detection level may seem low but given the limitations of detecting small peptides by mass spectrometry, this represents a substantial level of validation. Two translation events were validated by FLAG tagging transfection assay: LINC01116 and LINC00478 lncRNA-smORFs. The production of 2/45 lncRNA-smORF peptides is corroborated by previous studies in nonneuronal cells. *HAND2-AS1* (translated in Control and RA) is translated in human and rodent heart and encodes for an integral membrane component of the endoplasmic reticulum (van Heesch et al. 2019). *CRNDE*, which is only translated upon differentiation, encodes for a previously characterized nuclear peptide (CRNDEP) (Szafron et al. 2015). The translation of these smORFs in multiple cell types provides substantial support for the production of peptides and their potential function.

We also discovered that 24% of the lncRNA-smORFs we find translated show sequence conservation across *Hominidae*. This suggests that the other great apes have the potential to translate very similar peptides. This provides additional evidence to indicate that these translation events are not translational noise. Of course, it will be interesting to uncover the function of these small peptides in the future. Four of the conserved lncRNA-smORFs are under purifying selection and therefore likely to encode peptides.

*LINC01116*-smORF DNA sequence is on the opposite strand to a SINE element, suggesting that this lncRNA and smORF have likely evolved from a SINE transposable element (TE). This is consistent with previous observations that 39% of lncRNA sequences are derived from TEs (Carlevaro-Fita et al. 2016) and that *LINC01116* is human specific, that is, not found in other apes. Many other small ORFs have also been found to originate from Alu elements; for example, 287 human uORFs (Shen et al. 2011). Together this suggests that LINC01116-smORF has recently evolved from a TE.

We found that 22% of the translated lncRNAs are differentially expressed during neuronal differentiation of SH-SY5Y. Analysis of publicly available data sets revealed that our translated lncRNAs show regulated expression during human development, specifically in the brain, more so than lncRNAs in general. Many of these translated lncRNAs also exhibit associations with neuronal diseases. The FANTOM6 project data suggests that *LINC01116*, *FGD5-AS1*, and *TUG1* have cellular roles in neuronal function (Ramilowski et al. 2020). Together this indicates that these translated lncRNAs play roles in neuronal development and differentiation and likely contribute to neurological diseases.

Here we have discovered that *LINC01116* produces an 87aa peptide that exhibits cytoplasmic localization, and specifically is detected near the cell membrane and in neuritic processes. The up-regulation of *LINC01116* expression upon differentiation, coupled with the localization of its peptide, led us to further investigate its potential role in differentiation. Knockdown of *LINC01116* upon differentiation appears to impede neurite outgrowth and results in the reduction of the mRNA levels of the noradrenergic marker *MOXD1*. Our data suggest that *LINC01116* is involved in the regulation of neuronal differentiation, consistent with the fact that it is moderately expressed in the developing human forebrain and highly expressed in the developing human midbrain and spinal cord (Lindsay et al. 2016). The effects of siRNA in human dermal fibroblasts also supports a role for *LINC01116* in neuronal migration (Ramilowski et al. 2020). *LINC01116* has previously been found to be involved in two other cancer models: in the progression of glioblastoma (Brodie et al. 2017); and it is up-regulated in gefitinib resistant non-small cell lung cancer cells (Wang et al. 2020). siRNA knockdown of *LINC01116* in both these cell types results in decreased expression of stem-cell markers (*NANOG*, *SOX2* and *OCT4*) and reduced cell proliferation. This suggests *LINC01116* promotes cell proliferation in these systems, indicating that the downstream effects of *LINC01116* may vary according to cell type. However, knockdown of *LINC01116* also inhibited migration of glioma stem cells (Brodie et al. 2017), while overexpression of *LINC01116* promoted invasion and migration of gastric cancer cells (Su et al. 2019). This suggests a potential role of *LINC01116* in the formation of cell membrane protrusions, which is consistent with the role we have discovered for *LINC01116* in neurite development. It is yet to be determined if this function of *LINC01116* during neuronal differentiation is performed at the lncRNA or peptide level.

To conclude, our findings indicate that many lncRNAs are localized in the cytoplasm and they likely play functional roles as indicated by their regulation during differentiation and polysome association. Given the large number of lncRNAs we found to be associated with polysomes in the cytoplasm, it is likely that future work will assign the functions of many more lncRNAs to translational regulation. We have identified 43 novel translation events, many of which are regulated during differentiation. The lncRNA-smORFs we discover here represent a general population whose products have not yet been characterized. As demonstrated for *LINC01116*, lncRNAs and the small peptides encoded therein have the potential to contribute to important cellular functions, development, and disease.

## MATERIALS AND METHODS

### Cell culture

Human neuroblastoma SH-SY5Y cells, were cultured in Dulbecco's Modified Eagle Medium (DMEM 4.5g/L Glucose with l-Glutamine) supplemented with 1% (v/v) Penicillin/Streptomycin and 10% Fetal Bovine Serum (FBS) at 37°C, 5% CO_2_. Neural induction commenced at passage 4 and was performed as described previously (Korecka et al. 2013; Forster et al. 2016) with minor alterations. All trans Retinoic Acid (RA, Sigma) was added to cells 24 h after plating, at a final concentration of 30 µM for 3 d.

### Immunocytochemistry

Cells were seeded on Poly-d-Lysine/mouse laminin coated 12 mm round coverslips (Corning BioCoat Cellware) and fixed with 4% paraformaldehyde (PFA) (Affymetrix) for 20 min at room temperature (RT). A permeabilization step (0.1% Triton-X for 10 min at RT) was performed prior to blocking, followed blocking at RT in blocking buffer (3% BSA, 1× PBS or 5% NGS, 1× PBS and 0.1% Triton-X) for 30 min. Primary antibodies (Supplemental Methods) were applied in 3% BSA 1× PBS or 0.5% NGS, 1× PBS, 0.1% Triton-X and incubated at RT for 2 h or at 4°C overnight. Cells were washed and labeled with Alexa 488, Alexa 555, or Alexa 633 at 1:500 dilution for 2 h at RT in 0.5% NGS, 1× PBS, 0.1% Triton-X. Cells were mounted in VECTASHIELD mounting medium, analyzed using LSM 700 confocal microscope (Zeiss) ImageJ.

### cDNA synthesis and quantitative real time PCR (RT-qPCR)

Equal amounts of RNA (whole cell, nuclear, and cytoplasmic lysates) or equal volumes (polysome fractions) were subject to cDNA synthesis, using qScript (Quantabio) according to manufacturer's instructions. qPCR was performed using the CFX Connect Thermal Cycler and quantification using SYBR Green fluorescent dye (PowerUp SYBR Green Master Mix, Thermo Fisher Scientific). Primers were designed to anneal to exon–exon junctions, where possible, or to common exons between alternative transcripts (Supplemental Methods). Target mRNA and lncRNA levels were assessed by absolute quantification by the means of standard curve or relative quantification, using the ΔΔCq method.

### Polysome profiling

RA was added to SH-SY5Y cells 3 d prior to harvesting. Cells were treated with cycloheximide (Sigma) at 100 µg/mL for 3 min at 37°C, washed (1× PBS, 100 µg/mL cycloheximide) and trypsinized for 5 min at 37°C. Subsequently, cells were pelleted, washed (1× PBS, 100 µg/mL cycloheximide), and resuspended in ice cold lysis buffer (Supplemental Methods); 50 mM Tris-HCl pH 8, 150 mM NaCl, 10 mM MgCl_2_, 1 mM DTT, 1% IGEPAL, 100 µg/mL cycloheximide, Turbo DNase 24 U/µL (Invitrogen), RNasin Plus RNase Inhibitor 90U (Promega), cOmplete Protease Inhibitor (Roche), for 45 min. Cells were then subjected to centrifugation at 17,000*g* for 5 min, to pellet nuclei. Cytoplasmic lysates were loaded onto 18%–60% sucrose gradients (∼70 × 10^6^ cells per gradient) at 4°C and subjected to ultracentrifugation (121,355 × g_avg_ 3.5 h, 4°C) in SW-40 rotor. Gradients were fractionated using Gradient Station (Biocomp) and absorbance at 254 nm was monitored using a Bio-Rad detector.

### Poly-Ribo-Seq

Approximately 20% of cytoplasmic lysate was kept for poly(A) selection (total RNA control) and ∼80% was loaded onto 18%–60% sucrose gradients (∼70 × 10^6^ cells per gradient) at 4°C and subjected to ultracentrifugation (121,355 × g_avg_ 3.5 h, 4°C) in SW-40 rotor. Polysome fractions were pooled from control and from differentiated cells. Approximately 25% polysomes were kept for poly(A) selection (polysome-associated RNA). The remaining 75% was diluted in 100 mM Tris-HCl pH 8, 30 mM NaCl, 10 mM MgCl_2_. RNaseI (EN601, 10 U/µL 0.7–1 U/million cells) was subsequently added and incubated overnight at 4°C. RNaseI was deactivated using SUPERase inhibitor (200 U/gradient) for 5 min at 4°C. Samples were concentrated using 30 kDa molecular weight cutoff columns (Merck) and loaded on sucrose cushion (1 M sucrose, 50 mM Tris-HCl pH 8, 150 mM NaCl, 10 mM MgCl_2_, 40 U RNase Inhibitor) and subjected to ultracentrifugation at 204,428 × g_avg_ at 4°C for 4 h (TLA110). Pellets were resuspended in TRIzol (Ambion, Life Technologies) and processed for RNA purification.

RNA purification from cytoplasmic lysates and RNaseI footprinted samples was performed by TRIzol RNA extraction, following manufacturer's instructions. RNA purification from polysome fractions was performed by isopropanol precipitation, followed by TURBO DNase treatment (Thermo Fisher) (according to manufacturer's instructions), acidic phenol/chloroform RNA purification and ethanol precipitation at −80°C overnight. RNA concentration was determined by Nano-drop 2000 software. Two rounds of poly(A) selection from total cytoplasmic lysate and polysome fractions were performed using oligo (dT) Dynabeads (Invitrogen) according to manufacturer's instructions. Poly(A) RNA was fragmented by alkaline hydrolysis. A total of 28–34 nt ribosome footprints and 50–80 nt mRNA fragments were gel purified in 10% (w/v) polyacrylamide-TBE-Urea gel at 300 V for 3.5 h in 1× TBE. Ribosome footprints were subjected to rRNA depletion (Illumina RiboZero rRNA Removal Kit).

5′ stranded libraries were constructed using NEB Next Multiplex Small RNA Library Prep. The resulting cDNA was PCR amplified and gel purified prior to sequencing. Libraries were subjected to 75 bp single end RNA-seq using NextSeq500 Illumina sequencer, High Output Kit v2.5 (75 Cycles) (Next Generation Sequencing Facility, Faculty of Medicine, University of Leeds).

### RNA-seq data analysis

RNA-seq reads were trimmed with Cutadapt (v.19.1) (Martin 2011) and filtered with fastq_quality_filter (v.0.0.13) (Hannon 2010) to filter out the reads of low quality (90% of the reads to have a phred score above 20). Filtered reads were mapped (Liao et al. 2013) to the human genome reference (the lncRNA GENCODE Release 19 [Frankish et al. 2019] and annotation added to mRNA annotation from the UCSC [Haeussler et al. 2019] human genome assembly [hg19] from iGenomes) with Rsubread (v.1.22.0) (Liao et al. 2013), and uniquely mapped reads were reported. Bam file sorting and indexing was performed with SAMtools (v.1.3.1) (Li et al. 2009). Subsequently summarized read counts for all genes were calculated using featureCounts (Liao et al. 2014). For normalization, RPKM values were calculated. Differential expression analysis was conducted with DESeq2 (v.1.12.0) (Love et al. 2014) based on the two cutoffs *P*^adj^ < 0.05 and the absolute value of log_2_FoldChange > 1. Gene ontology analysis was performed with GOrilla (Gene Ontology enRIchment anaLysis and visuaLizAtion tool) (Eden et al. 2009).

### Ribo-Seq analysis

Quality reports of polysome-associated RNA-seq and Ribo-Seq data were made using Fastqc (v.0.11.9) (Andrews 2010). Adaptor sequences were trimmed using Cutadapt (v.210) (Martin 2011) with a minimum read length of 25 bp, and untrimmed outputs retained for RNA-seq reads. Low-quality reads (score <20 for 10% or more of reads) were then discarded using FASTQ Quality Filter, FASTX-Toolkit (v.0.0.14) (Gordon 2010). Human rRNA sequences were retrieved from RiboGalaxy (Michel et al. 2016) and high confidence hg38 tRNA sequences from GtRNAdb Release 17 (Chan and Lowe 2016). One base was removed from the 3′ ends of reads to improve alignment quality; reads originating from rRNA and tRNA were aligned and removed using Bowtie2 (v.2.4.1) (Langmead and Salzberg 2012).

The splice aware aligner STAR (v2.7.5c) (Dobin et al. 2012) was used to map remaining reads to the human reference genome (GRCh38.p12), GENCODE release 30 (Frankish et al. 2019). The STAR (v2.7.5c) (Dobin et al. 2012) genome index was built with a sjdbOverhang of 73. SAMtools (v.1.10) (Li et al. 2009) was used to create sorted, indexed bam files of the resulting alignments.

Metaplots of aligned Ribo-Seq data were generated using metaplots.bash script from Ribotaper (v1.3) (Calviello et al. 2016) pipeline. These show the distance between the 5′ ends of Ribo-Seq and annotated start and stop codons from CCDS ORFs, allowing the locations of P-sites to be inferred. Read lengths exhibiting the best triplet periodicity were selected for each replicate, along with appropriate offsets (Supplemental Fig. 5; Supplemental Table 1).

Actively translated smORFs were then identified using Ribotaper (v1.3) (Calviello et al. 2016). Initially, this requires an exon to contain more than five P-sites in order to pass to quality control steps. Identified ORFs were then required to have a 3-nt periodic pattern of Ribo-Seq reads, with 50% or more of the P-sites in-frame. In the case of multiple start codons, the most upstream in-frame start codon with a minimum of five P-sites in between it and the next ATG was selected. ORFs for which >30% of the Ribo-Seq coverage was only supported by multimapping reads were also subsequently filtered. For a smORF to be considered actively translated in a condition, we required that it be identified in at least two of the three biological replicates for the condition.

Specific metaplots were also created for the 45 translated lncRNA-smORFs, and 100 randomly selected translated ccds ORFs, to compare ribosome enrichment around the start and stop codons in our protocol. P-sites were computed for each position in a 75 nt window around the start and stop codons, and scaled by the total number of reads in the two windows for each transcript. The mean normalized counts were then taken for each position in the two windows and plotted.

Translational efficiency (TE) was estimated for all translated ORFs in each condition, where TE was equal to the mean number of P sites per ORF, normalized by the median P sites per ORF per replicate, divided by the mean number of RNA sites per ORF, normalized by the median RNA sites per ORF per replicate.

### smORF peptide analysis

For each of our ORF sets (protein coding, lncRNA-smORF, uORF, and dORFs), the average amino acid compositions were calculated. Random control expected frequencies were taken from King and Jukes (King and Jukes 1969).

Two published SH-SY5Y cell mass proteomics data sets were analyzed: PXD010776 (Murillo et al. 2018) and PXD014381 (Brenig et al. 2020). Binary raw files (*.raw) were downloaded from PRIDE then converted to human-readable MGF format using ThemoRawFileParser (Hulstaert et al. 2020). The amino acid sequences of our translated uORFs, dORFs, and lncRNA-smORFs were added to the whole *Homo sapiens* proteome data set (20,379 entries) downloaded from UniProtKB (Bateman et al. 2019) in November 2019. The new FASTA file was then used as a customized database on Comet (v2019.01.2) (Eng et al. 2013) search engine runs that scanned all MS/MS files (*.mgf) against it.

Default settings in Comet were used with the following exceptions according to the MS/MS data type. iTRAQ-4plex (PXD010776): decoy_search = 1, peptide_mass_tolerance = 10.00, fragment_bin_tol = 0.1, fragment_bin_offset = 0.0, theoretical_fragment_ions = 0, spectrum_batch_size = 15000, clear_mz_ range = 113.5–117.5, add_Nterm_peptide = 144.10253, add_K_lysine = 144.10253, minimum_peaks = 8. Label-free (PXD014381): decoy_search = 1, peptide_mass_tolerance = 10.00, fragment_ bin_tol = 0.02, fragment_bin_offset = 0.0, theoretical_fragment_ions = 0, spectrum_batch_size = 15000. CometUI (Eng et al. 2013) was used for analyzing MS/MS data and setting a false discovery rate (FDR) threshold of 10% per peptide identification. This FDR threshold was selected due to expected low abundance levels of the target smORFs.

### Conservation analysis

Protein, cDNA, and ncRNA sequence data for *H. sapiens*, *P. abelii*, *P. paniscus*, *P. troglodytes*, *G. gorilla*, *N. leucogenys*, and *M. musculus* were obtained from Ensembl (release 100 [Yates et al. 2020]). LncRNAs are poorly conserved so we selected five species of apes with well-annotated genomes (four of these are great apes), and *M. musculus* represents an outgroup. These data formed the subject database for subsequent homology searches.

A number of criteria were considered to deem an ORF “conserved.” At the protein level, these included a pairwise distance of 50% or less, syntenous positions in the genome, and the finding of ortholog groups exhibiting similar conservation to the human ORF. At the transcript level (using cDNA and ncRNA data), the above criteria were considered, along with the conservation of a start codon and subsequent sequence. If the same results were returned multiple times by the search strategies described below, they were also given extra consideration. In all cases the focus was on the conservation of the ORF sequence, not necessarily the surrounding transcript.

Sequence homology searches were performed using BLASTp (e-value = 0.001) where the 45 translated human lncRNA peptide sequences formed the queries and the protein sequences for *P. abelii*, *P. paniscus*, *P. troglodytes*, *G. gorilla*, *N. leucogenys*, and *M. musculus* formed the subject database (Altschul et al. 1990). Results were filtered to remove anything with <75% identity, unless a result(s) was the lowest e-value hit for a given query in each species. Results were returned for 12 lncRNA peptides, and these were manually cross-validated using the Ensembl Genome Browser and multiple sequence alignments generated in ClustalOmega (Sievers et al. 2011). Default parameter settings were applied in the msa package in R (Bodenhofer et al. 2015).

The transcript sequences of the 45 translated lncRNAs were searched against transcriptome databases created by combining the cDNA and ncRNA data for each species, using BLASTn (e-value = 0.001) (Altschul et al. 1990). Results of this BLAST were used to filter the initial BLAST databases. ORF portions of the 45 translated lncRNAs were extracted and searched against these filtered databases using BLASTn (e-value = 0.001) (Altschul et al. 1990).

The homology searches confirmed the genes of origin for all 45 lncRNA-smORFs in *H. sapiens* at the nucleotide and peptide level. For the nucleotide sequence searches, the remaining species could identify homologs for 18 of the lncRNA ORF queries. These were cross-validated as described above (i.e., manually and using MSAs), resulting in 14 lncRNA-smORFs with evidence of sequence conservation based on transcript sequences. For the protein sequences, the remaining species returned result homologs for 21 of the lncRNA peptide queries. As some queries had many spurious results, they were further filtered to select the transcripts(s) with the lowest e-value for each query in each species. These were cross validated as above, resulting in 16 lncRNA peptides with evidence of sequence conservation based on transcript sequences. We combined evidence from both approaches into a final data set consisting of 17 lncRNA-smORFs with evidence of conservation in at least one of the six species queried.

The nucleotide alignments of the 17 lncRNA-smORFs were manually curated and trimmed, and evaluated using the 58 mammals model in PhyloCSF (Lin et al. 2011). As Bonobo is not in this model, three sequences could not be evaluated, and the putative Bonobo ORF was removed from a further three alignments.

### Cytoplasmic/nuclear fractionation of SH-SY5Y cells

Cells were harvested and washed with 1X PBS. Cells were lysed in whole-cell lysis buffer (Supplemental Methods) (500 µL buffer per 10^6^ cells) on ice for 30 min. Whole cell lysate aliquots were removed and remainder subjected to centrifugation at 1,600*g* for 8 min to pellet nuclei. Nuclear and cytoplasmic fractions were subjected to two further clearing steps by centrifugation (3000*g* and 10,000*g*, respectively). Nuclei were lysed in RIPA buffer (Supplemental Methods). Approximately 10% of both nuclear and cytoplasmic lysates were used for western blot and ∼90% subjected to RNA extraction (ZYMO R1055).

### Western blot

Samples were diluted in 4× Laemmli sample buffer (Bio-Rad) (277.8 mM Tris-HCI, pH 6.8, 4.4% LDS, 44.4% (v/v) glycerol, 0.02% bromophenol blue), 5% β-mercaptoethanol (Sigma) was added prior to heating at 95°C for 5 min and loaded on 10% SDS gels. Gel electrophoresis was performed using the Bio-Rad Mini-PROTEAN 3 gel electrophoresis system (Bio-Rad Laboratories). Proteins were transferred to nitrocellulose membranes (Amersham Protran) and blocked with 5% fat-free milk powder in 1× PBS, 0.05% Tween-20 (Sigma) for 1 h at RT. Blots were incubated with primary antibodies overnight (Table 1). Blots were then washed in PBS-T and incubated with secondary antibody (anti-mouse HRP) at RT for 2 h. Membranes were washed three times with PBS-T, prior to application of ECL (Biological Industries). Chemiluminescent signal was detected with Chemi-Doc (Bio-Rad). All membranes were probed for β-tubulin as loading control.

### Analysis of publicly available lncRNA data

Dynamic differential expression analysis data were accessed through lncExpDB (Cardoso-Moreira et al. 2019; Sarropoulos et al. 2019), where R package maSigPro was used to identify developmentally dynamically lncRNAs, that is, genes that show large changes in expression during development of a specific organ. Thirty-two lncRNA genes identified by Poly-Ribo-Seq as translated were present in lncExpDB. Disease association analysis used data from lncRNADisease (Chen et al. 2013; Bao et al. 2019), Differential Expression Atlas (https://www.ebi.ac.uk/gxa/experiments?species=homo%20sapiens), Cancer RNA-seq Nexus (Li et al. 2016), Malacards (Rappaport et al. 2013, 2014, 2017). Genes were considered related to a disease if they showed significant differential expression between diseased and control conditions (log_2_ fold > 1, *P* < 0.05) or if they had been experimentally validated in the literature.

### smORF tagging

5′-UTRs and CDSs of putative smORFs (lacking stop codon) were generated by PCR (Supplemental Methods), using NEB High Fidelity DNA Polymerase (Q5). Carboxy-terminal 3×FLAG tag was incorporated within the reverse primer (Supplemental Methods) by PCR and products were cloned into NheI and EcoRV restriction sites (Supplemental Methods) of pcDNA3.1/Hygro Vector (Addgene, kindly provided by Mark Richards-Bayliss group, University of Leeds). Start codon mutations were generated by site directed mutagenesis (Q5 Site Directed Mutagenesis Kit, NEB).

Plasmid transfections were performed using Lipofectamine 3000 (Thermo) following the manufacturer's instructions. After 48 h, the cells were fixed for 20 min with 4% paraformaldehyde, washed with 1× PBS, 0.1% Triton X-100 (PBS-T) and processed for immunocytochemistry as previously described. Imaging was conducted using EVOS fluorescent microscope.

### siRNA knockdown

siRNA knockdown was performed using Lincode siRNA SMARTpool (Dharmacon) (LINC01116 transcript: R-027999-00-0005 SMARTpool). Lincode Non-targeting Pool (D-001810-10) was used as scrambled control. Cells were seeded in 24-well plates (10^5^ cells/well,) and siRNA were transfected using RNAiMAX lipofectamine (Thermo Fisher) as per manufacturer's instructions.

### General statistics and plots

Statistical analyses were performed in R (R Core Team 2019), using packages including stringr (Wickham 2019), dplyr (Wickham et al. 2017), tidyr (Wickham 2017), protr (Xiao et al. 2015) ggplot2 (Wickham 2016), ggstatsplot (Patil 2018), knitr (Xie 2020), seqinr (Charif and Lobry 2007), ggbeeswarm (Clarke and Sherrill-Mix 2017), and EnhancedVolcano (Blighe et al. 2018).

Experimental values (RT-qPCR, are under polysome graphs, % of cells) from independent samples with equal variances were assessed using two-tailed unpaired Student's *t*-test. The results are shown as mean ± SEM values of three independent replicates. The exact *P*-values are described and specified in each figure legend. *P*-values <0.05 were considered statistically significant.

## DATA DEPOSITION

Poly-Ribo-Seq data sets have been deposited in GEO with ID GSE166214.

## SUPPLEMENTAL MATERIAL

Supplemental material is available for this article.

## Supplementary Material

Supplemental Material
