# Supplementary material for: Cytoplasmic long noncoding RNAs are differentially regulated and translated during human neuronal differentiation
Source: RNA. 2021 Sep;27(9):1082–101. doi: 10.1261/rna.078782.121 (PMC8370745; doi:10.1261/rna.078782.121)
Supplement: Supplemental Material [file supp_27_9_1082__DC1.html]

Cytoplasmic long noncoding RNAs are differentially regulated and translated during human neuronal differentiation — Supplemental Material 

# Cytoplasmic long noncoding RNAs are differentially regulated and translated during human neuronal differentiation

## Supplemental Material

- Supplemental\_Figures\_S1-S9\_Table\_1.pptx
- Supplemental\_Methods.docx
- Supplemental\_Table\_2.xlsx
