# Supplementary material for: Cytoplasmic long noncoding RNAs are differentially regulated and translated during human neuronal differentiation
Source: RNA. 2021 Sep;27(9):1082–101. doi: 10.1261/rna.078782.121 (PMC8370745; doi:10.1261/rna.078782.121)
Supplement: Supplemental Material [file supp_078782.121_Supplemental_Figures_S1-S9_Table_1.pptx]

## Slide 1
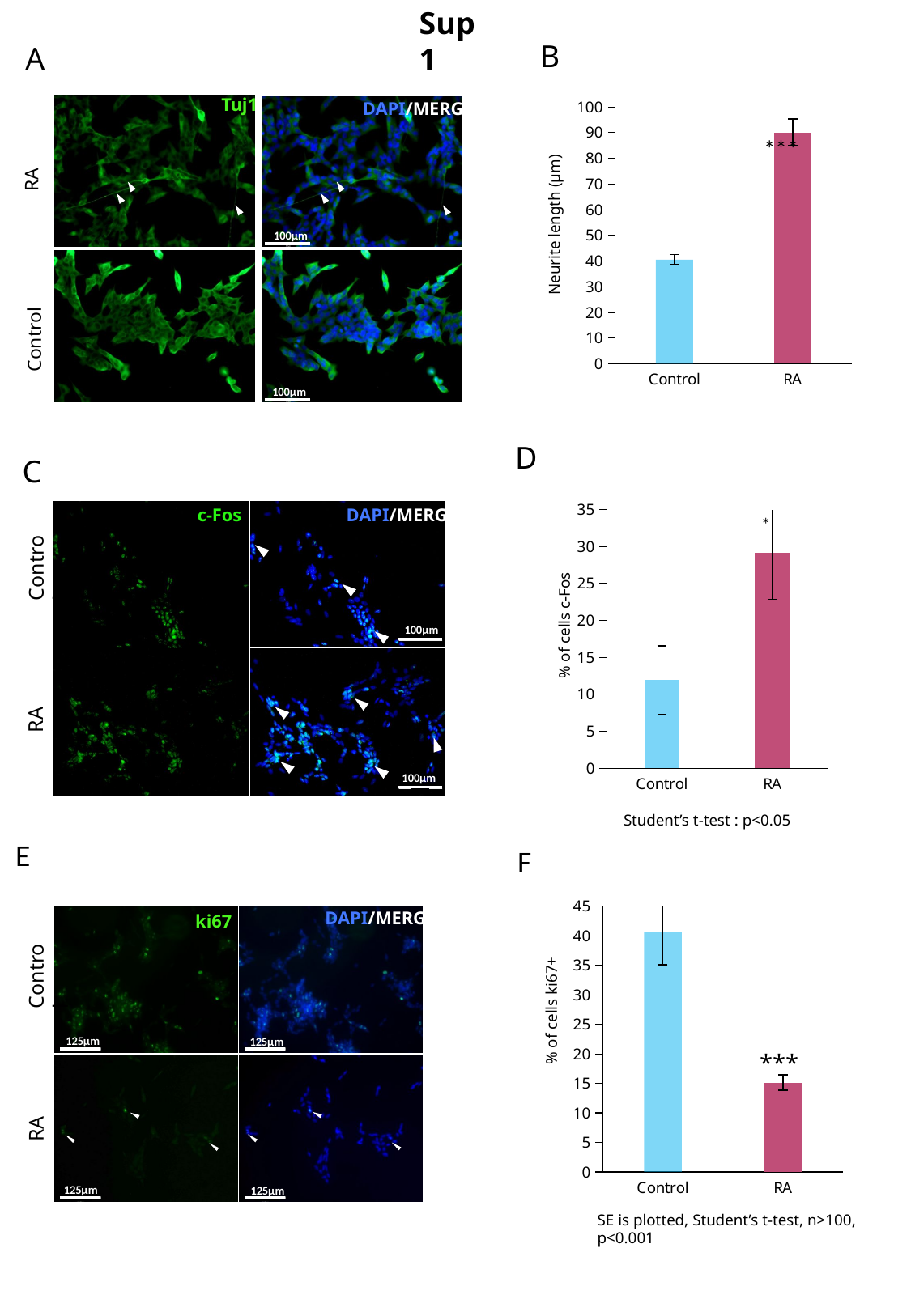

Sup 1
B
A
Tuj1
DAPI/MERGE
RA
100µm
Control
100µm
### Chart
| Category | neurite length(µm) |
|---|---|
| Control | 40.52549113300494 |
| RA | 90.11736380952378 |***
Neurite length (µm)
D
C
### Chart
| Category | %c-Fos+ |
|---|---|
| Control | 11.906400550585 |
| RA | 29.19303797468354 |% of cells c-Fos
Student’s t-test : p<0.05
c-Fos
DAPI/MERGE
Control
RA
100µm
100µm
*
E
F
### Chart
| Category | %ki67+ |
|---|---|
| Control | 40.66615027110767 |
| RA | 15.1241728967947 |% of cells ki67+
SE is plotted, Student’s t-test, n>100, p<0.001
***
DAPI/MERGE
ki67
Control
RA
125µm
125µm
125µm
125µm

## Slide 2
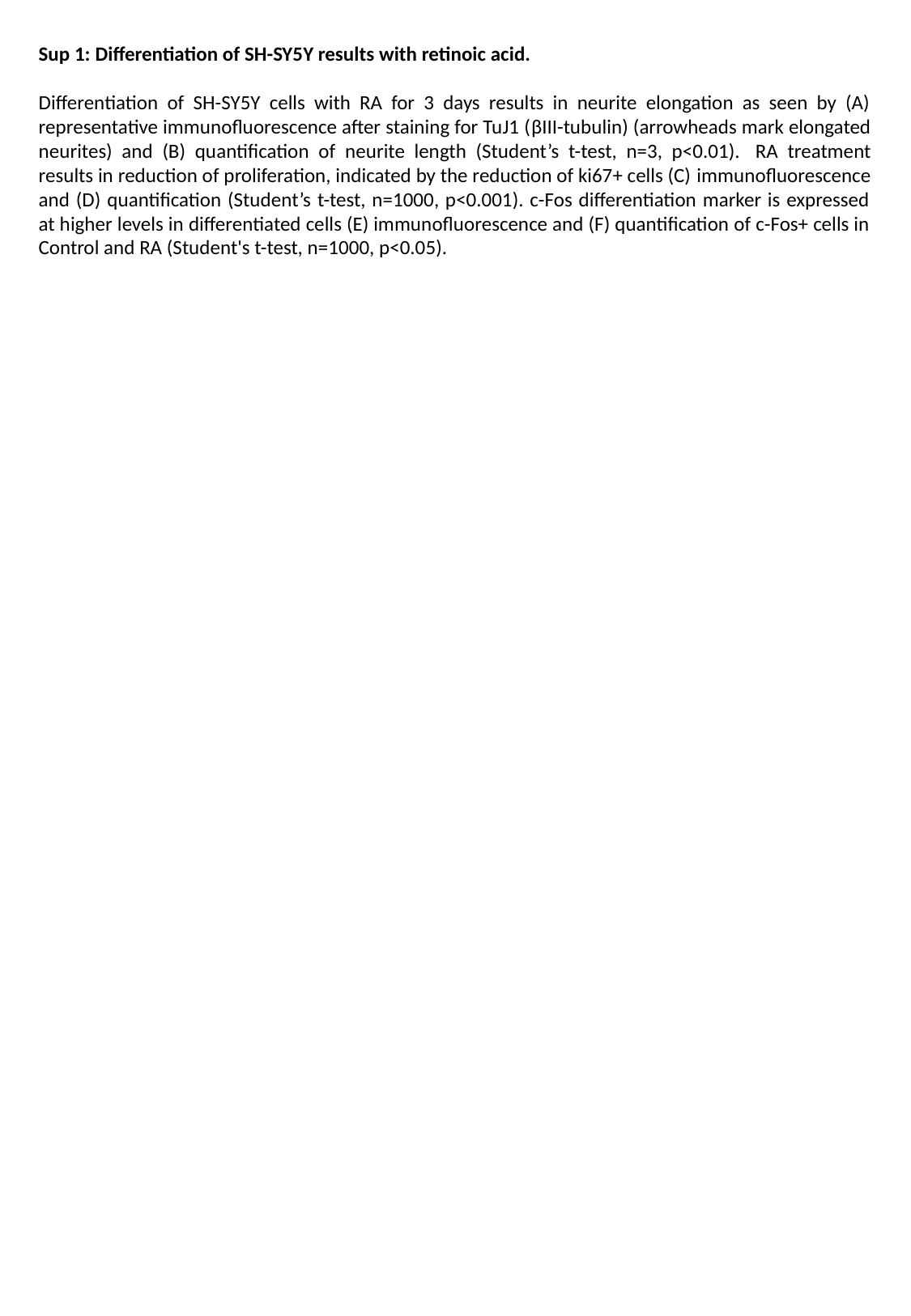

Sup 1: Differentiation of SH-SY5Y results with retinoic acid.
Differentiation of SH-SY5Y cells with RA for 3 days results in neurite elongation as seen by (A) representative immunofluorescence after staining for TuJ1 (βIII-tubulin) (arrowheads mark elongated neurites) and (B) quantification of neurite length (Student’s t-test, n=3, p<0.01).  RA treatment results in reduction of proliferation, indicated by the reduction of ki67+ cells (C) immunofluorescence and (D) quantification (Student’s t-test, n=1000, p<0.001). c-Fos differentiation marker is expressed at higher levels in differentiated cells (E) immunofluorescence and (F) quantification of c-Fos+ cells in Control and RA (Student's t-test, n=1000, p<0.05).

## Slide 3
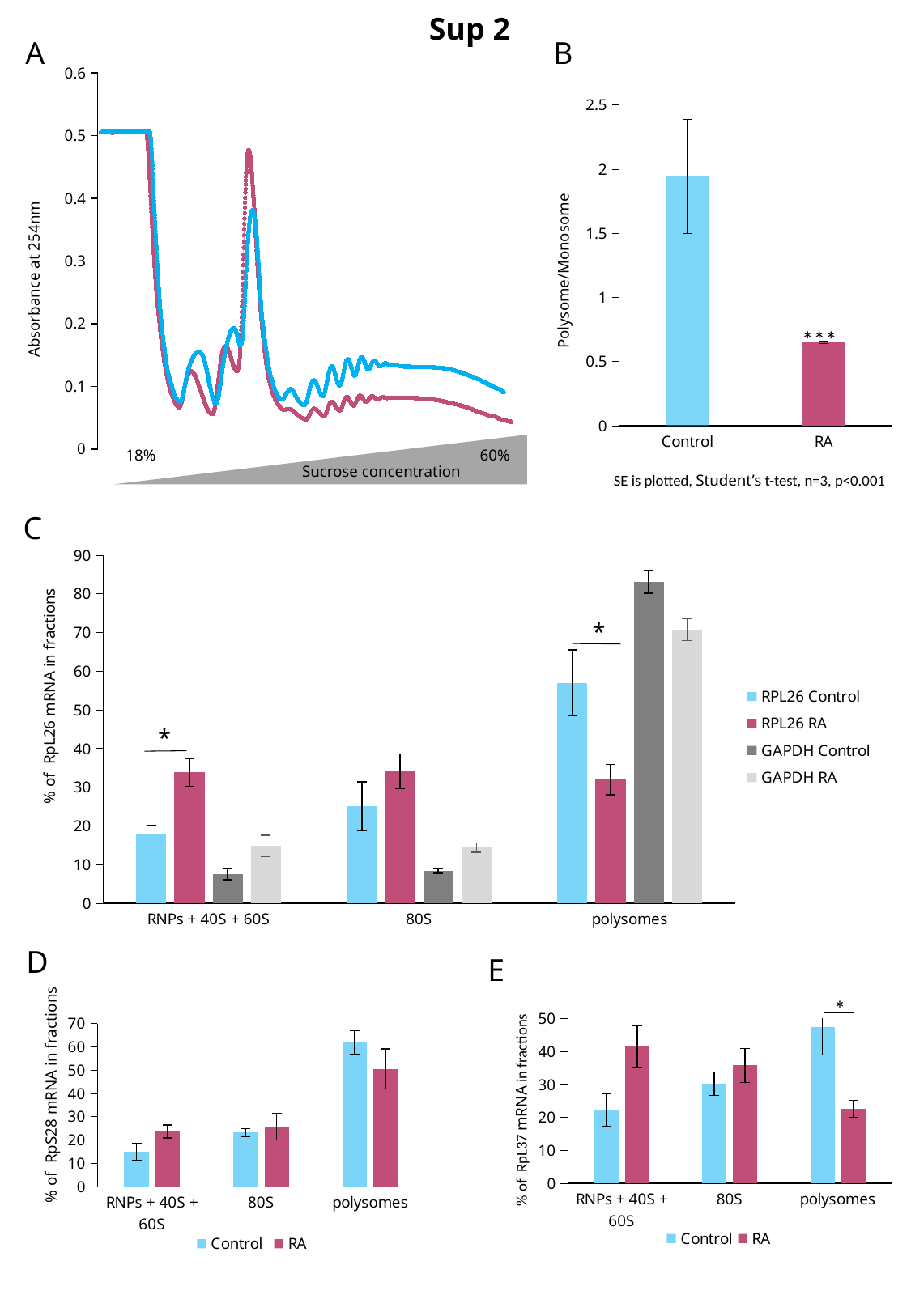

Sup 2
A
B
### Chart
| Category | CONTROL2 | RA30µM-4 |
|---|---|---|Absorbance at 254nm
18%
60%
Sucrose concentration
### Chart
| Category | P/M |
|---|---|
| Control | 1.945407681769183 |
| RA | 0.651482748286945 |Polysome/Monosome
***
SE is plotted, Student’s t-test, n=3, p<0.001
### Chart
| Category | RPL26 | RPL26 | GAPDH | GAPDH |
|---|---|---|---|---|
| RNPs + 40S + 60S | 17.85747973188063 | 33.83176692504387 | 7.541866717188547 | 14.82740823989111 |
| 80S | 25.1340762990624 | 34.15075997991576 | 8.36529917509935 | 14.36848600876043 |
| polysomes | 57.00844396905696 | 32.01747309504029 | 83.09420352965962 | 70.80410575134859 |*
% of RpL26 mRNA in fractions
*
E
C
D
### Chart
| Category | Control | RA |
|---|---|---|
| RNPs + 40S + 60S | 22.35837528205343 | 41.54241174948788 |
| 80S | 30.24114989980766 | 35.77395767571734 |
| polysomes | 47.40047481813891 | 22.68363057479472 |*
### Chart
| Category | Control | RA |
|---|---|---|
| RNPs + 40S + 60S | 14.89982144829915 | 23.70427803260746 |
| 80S | 23.33012207626308 | 25.7687441495985 |
| polysomes | 61.77005647543776 | 50.52697781779406 |% of RpS28 mRNA in fractions
% of RpL37 mRNA in fractions

## Slide 4
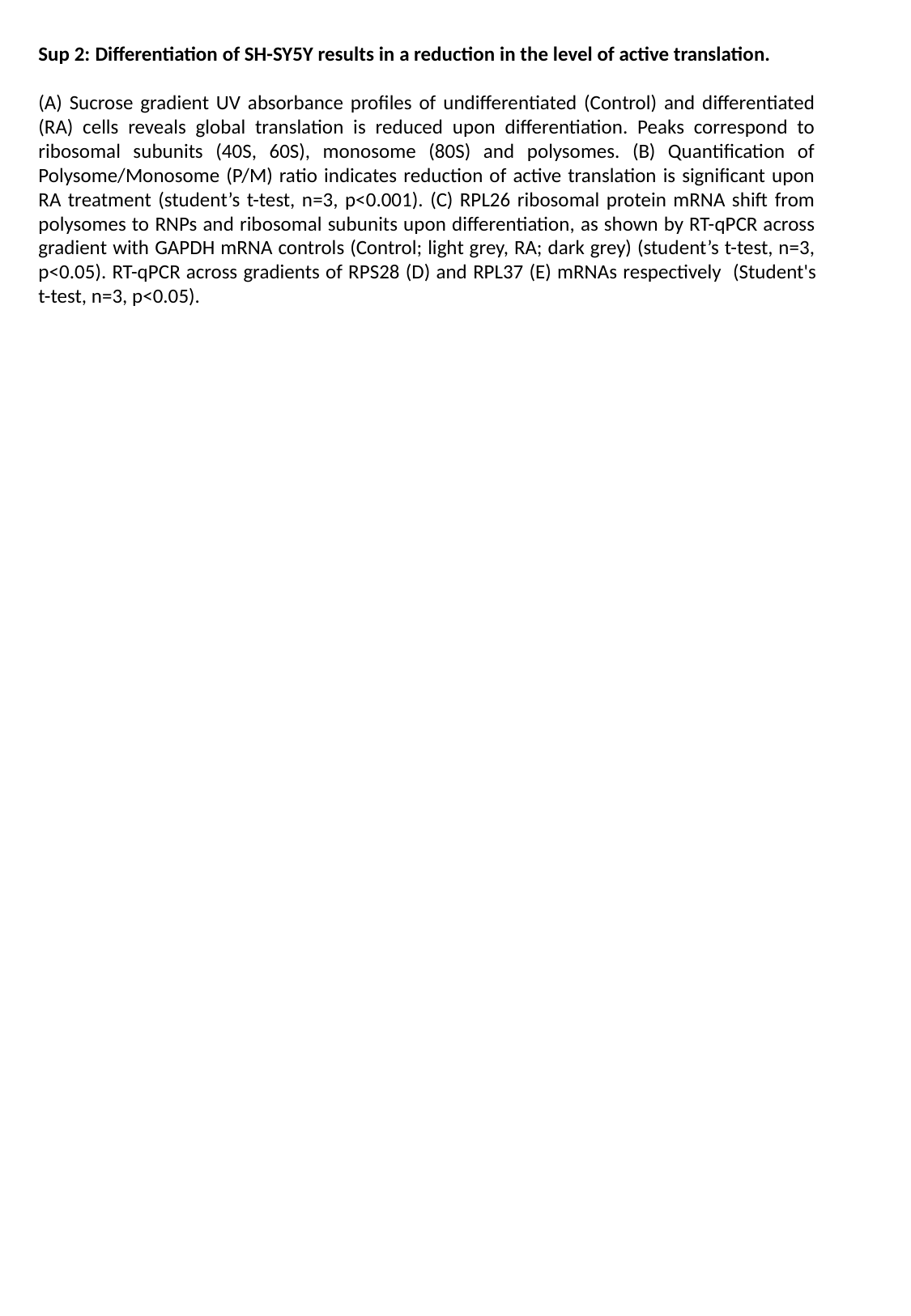

Sup 2: Differentiation of SH-SY5Y results in a reduction in the level of active translation.
(A) Sucrose gradient UV absorbance profiles of undifferentiated (Control) and differentiated (RA) cells reveals global translation is reduced upon differentiation. Peaks correspond to ribosomal subunits (40S, 60S), monosome (80S) and polysomes. (B) Quantification of Polysome/Monosome (P/M) ratio indicates reduction of active translation is significant upon RA treatment (student’s t-test, n=3, p<0.001). (C) RPL26 ribosomal protein mRNA shift from polysomes to RNPs and ribosomal subunits upon differentiation, as shown by RT-qPCR across gradient with GAPDH mRNA controls (Control; light grey, RA; dark grey) (student’s t-test, n=3, p<0.05). RT-qPCR across gradients of RPS28 (D) and RPL37 (E) mRNAs respectively  (Student's t-test, n=3, p<0.05).

## Slide 5
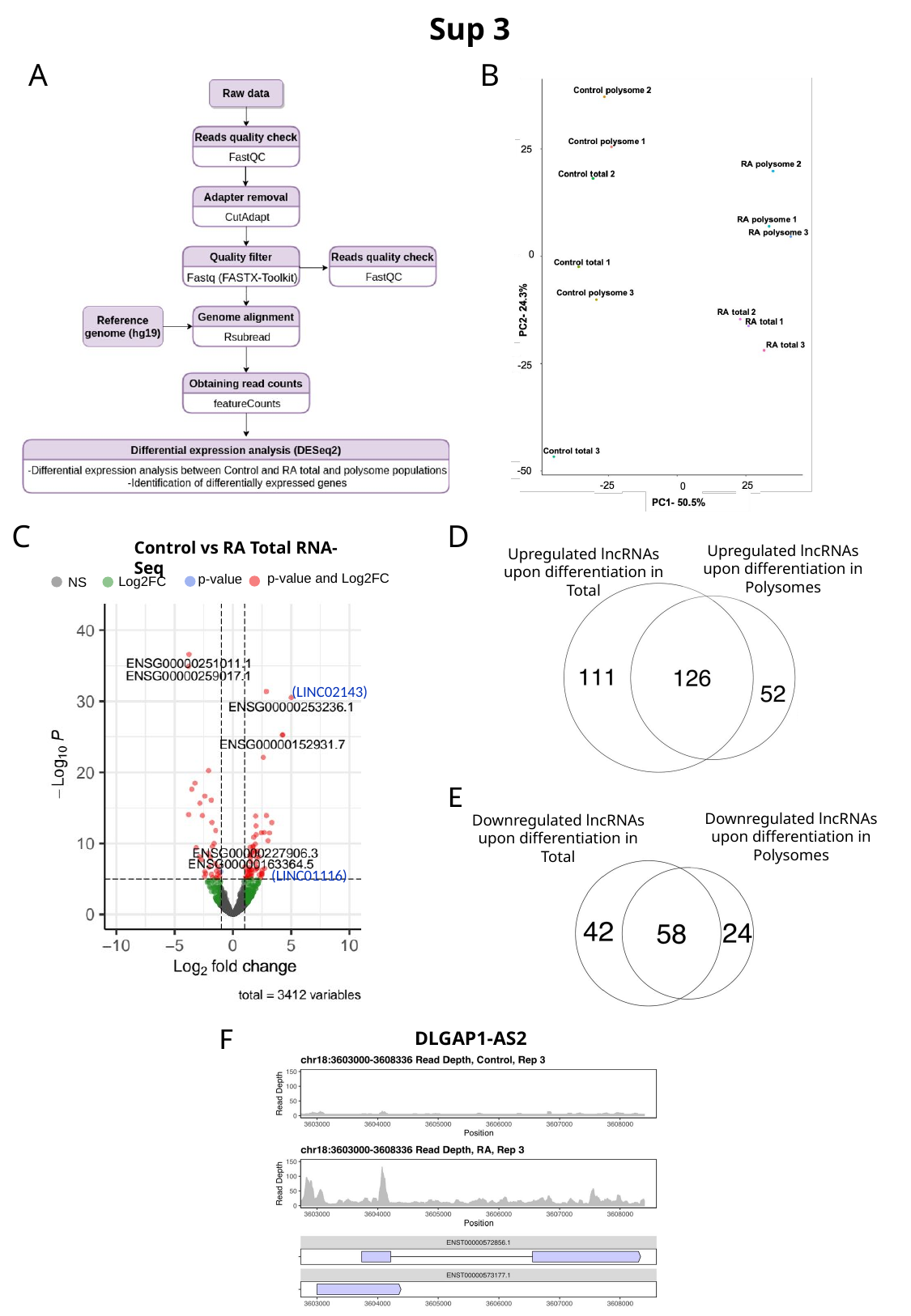

Sup 3
A
B
C
D
Control vs RA Total RNA-Seq
Downregulated
Upregulated
100
178
Upregulated lncRNAs upon differentiation in Polysomes
Upregulated lncRNAs upon differentiation in Total
p-value and Log2FC
p-value
Log2FC
NS
(LINC02143)
(LINC01116)
E
Downregulated lncRNAs upon differentiation in Polysomes
Downregulated lncRNAs upon differentiation in Total
E
F
 DLGAP1-AS2

## Slide 6
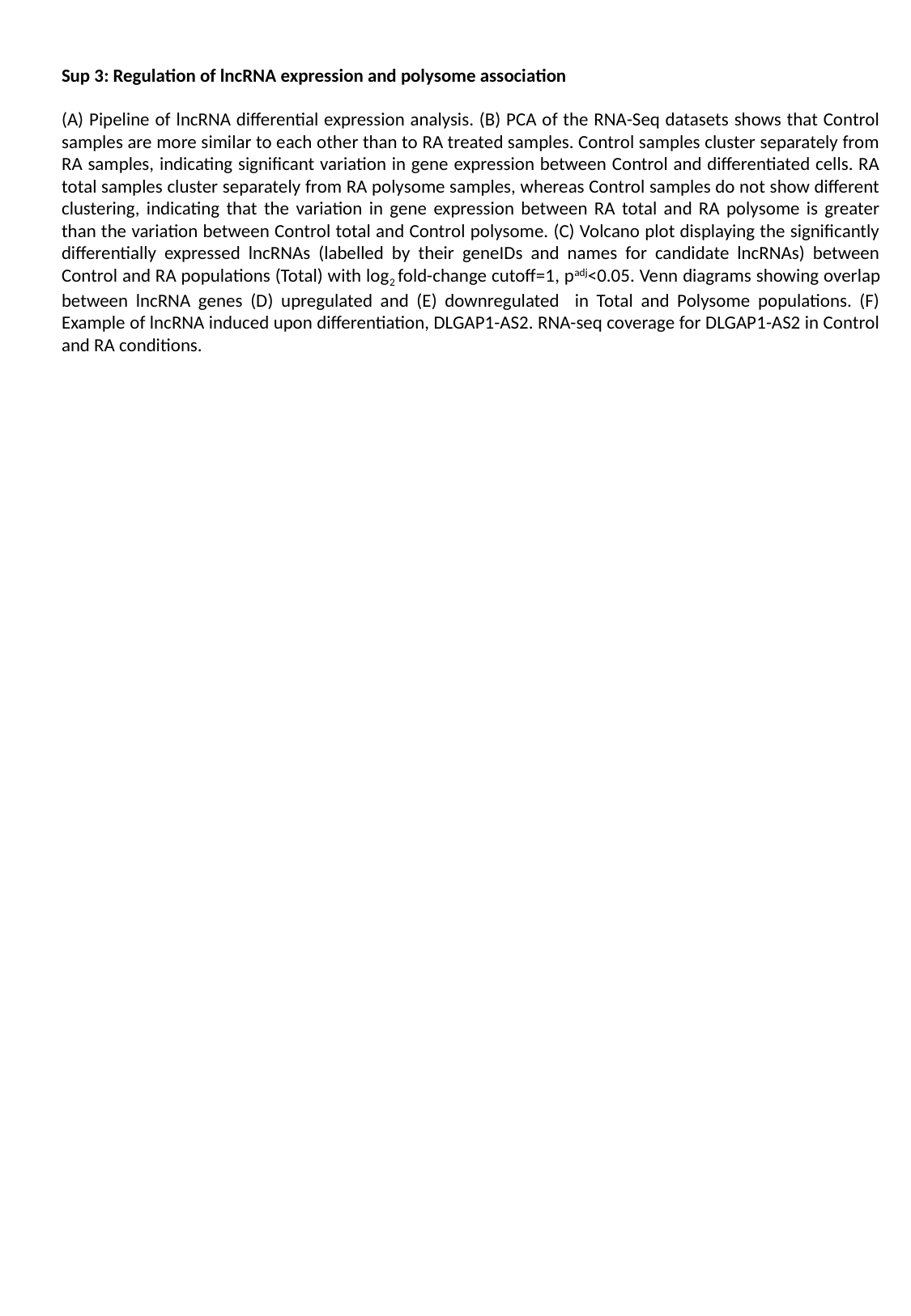

Sup 3: Regulation of lncRNA expression and polysome association
(A) Pipeline of lncRNA differential expression analysis. (B) PCA of the RNA-Seq datasets shows that Control samples are more similar to each other than to RA treated samples. Control samples cluster separately from RA samples, indicating significant variation in gene expression between Control and differentiated cells. RA total samples cluster separately from RA polysome samples, whereas Control samples do not show different clustering, indicating that the variation in gene expression between RA total and RA polysome is greater than the variation between Control total and Control polysome. (C) Volcano plot displaying the significantly differentially expressed lncRNAs (labelled by their geneIDs and names for candidate lncRNAs) between Control and RA populations (Total) with log2 fold-change cutoff=1, padj<0.05. Venn diagrams showing overlap between lncRNA genes (D) upregulated and (E) downregulated in Total and Polysome populations. (F) Example of lncRNA induced upon differentiation, DLGAP1-AS2. RNA-seq coverage for DLGAP1-AS2 in Control and RA conditions.

## Slide 7
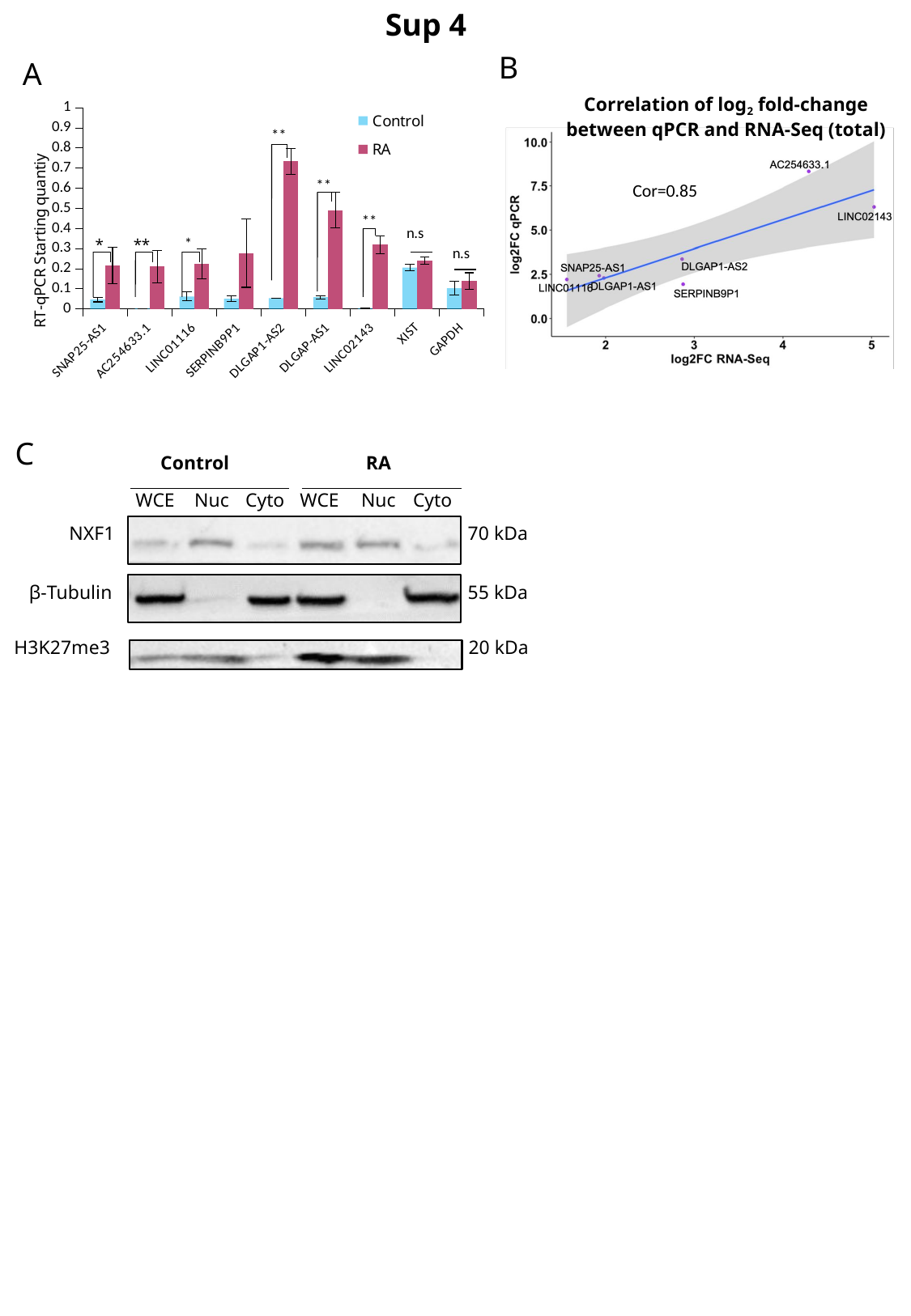

Sup 4
B
A
### Chart
| Category | Control | RA |
|---|---|---|
| SNAP25-AS1 | 0.046772097 | 0.214741196666667 |
| AC254633.1 | 0.00272498918462468 | 0.211160303592597 |
| LINC01116 | 0.0623314152884307 | 0.223310921175085 |
| SERPINB9P1 | 0.051450917600604 | 0.278692139760981 |
| DLGAP1-AS2 | 0.0537623094447606 | 0.733668736537438 |
| DLGAP-AS1 | 0.0586640725627066 | 0.491597271108067 |
| LINC02143 | 0.00338223836810204 | 0.319422955516298 |
| XIST | 0.205553867237425 | 0.240318202713587 |
| GAPDH | 0.103380883072041 | 0.138443390840553 |*
**
Correlation of log2 fold-change between qPCR and RNA-Seq (total)
Cor=0.85
C
Control
RA
WCE
Nuc
Cyto
WCE
Nuc
Cyto
NXF1
β-Tubulin
H3K27me3
70 kDa
55 kDa
20 kDa

## Slide 8
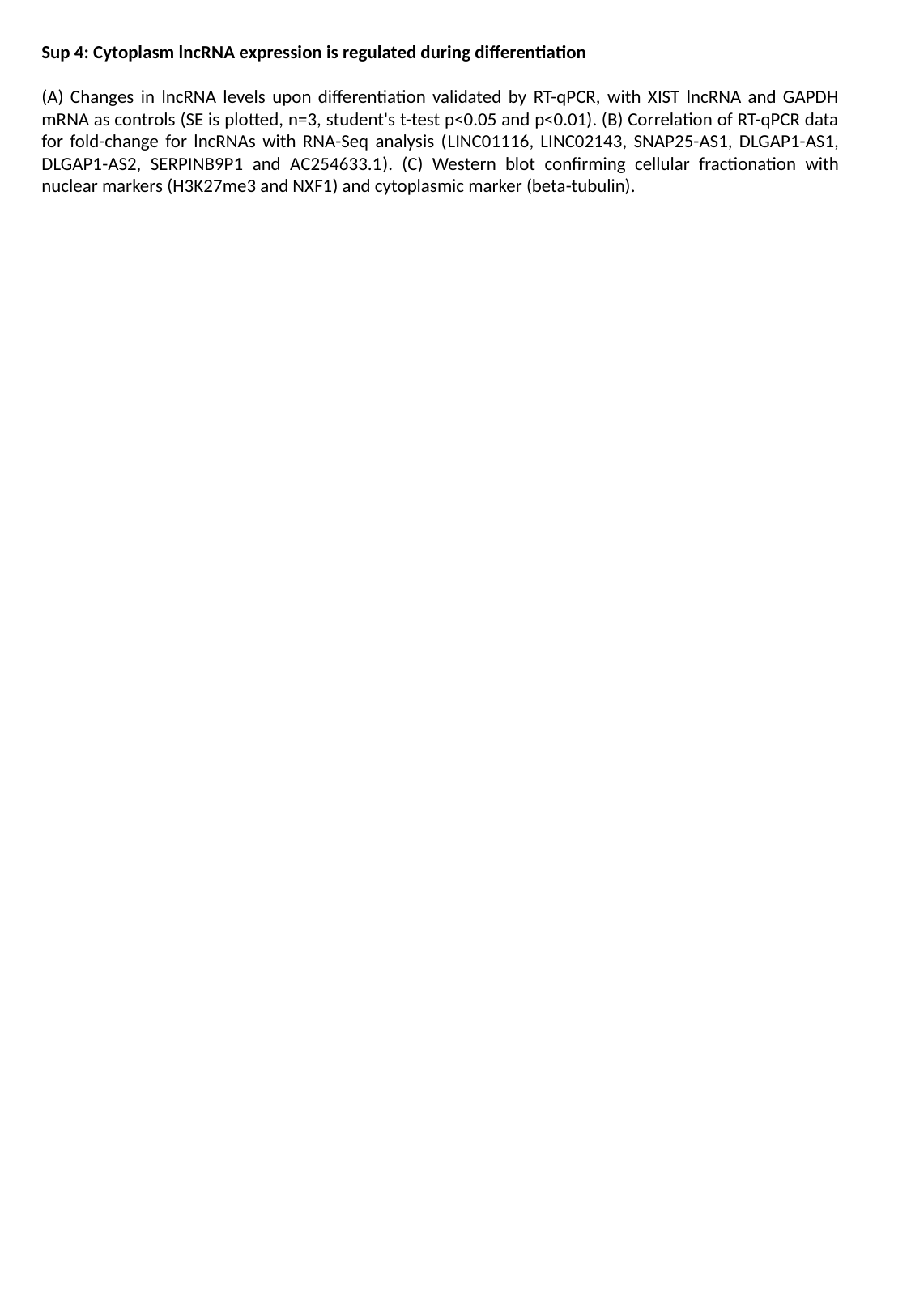

Sup 4: Cytoplasm lncRNA expression is regulated during differentiation
(A) Changes in lncRNA levels upon differentiation validated by RT-qPCR, with XIST lncRNA and GAPDH mRNA as controls (SE is plotted, n=3, student's t-test p<0.05 and p<0.01). (B) Correlation of RT-qPCR data for fold-change for lncRNAs with RNA-Seq analysis (LINC01116, LINC02143, SNAP25-AS1, DLGAP1-AS1, DLGAP1-AS2, SERPINB9P1 and AC254633.1). (C) Western blot confirming cellular fractionation with nuclear markers (H3K27me3 and NXF1) and cytoplasmic marker (beta-tubulin).

## Slide 9
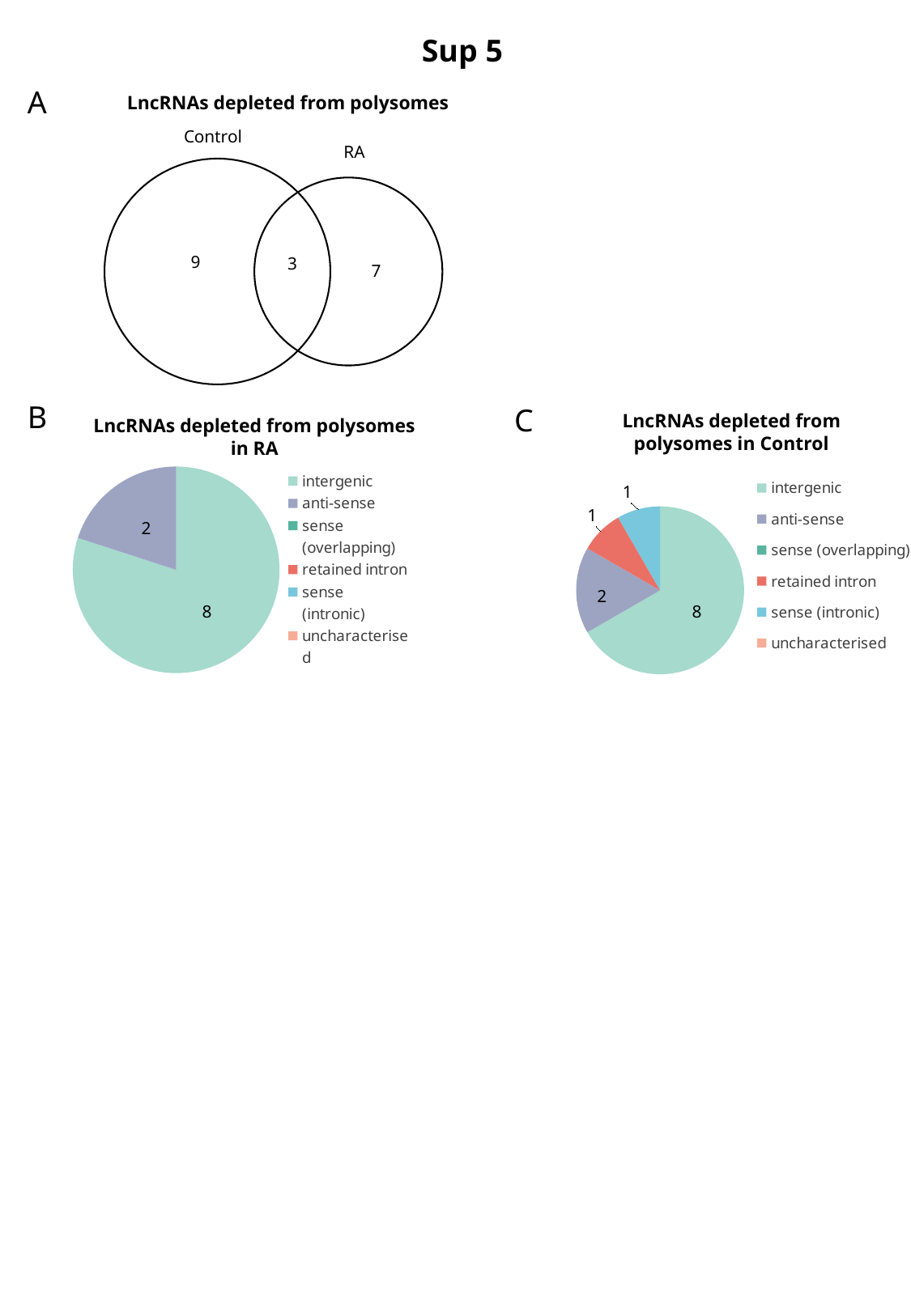

Sup 5
A
LncRNAs depleted from polysomes
Control
RA
9
3
7
B
C
LncRNAs depleted from polysomes in Control
LncRNAs depleted from polysomes in RA
### Chart
| Category | |
|---|---|
| intergenic | 8.0 |
| anti-sense | 2.0 |
| sense (overlapping) | 0.0 |
| retained intron | 1.0 |
| sense (intronic) | 1.0 |
| uncharacterised | 0.0 |
### Chart
| Category | |
|---|---|
| intergenic | 8.0 |
| anti-sense | 2.0 |
| sense (overlapping) | 0.0 |
| retained intron | 0.0 |
| sense (intronic) | 0.0 |
| uncharacterised | 0.0 |

## Slide 10
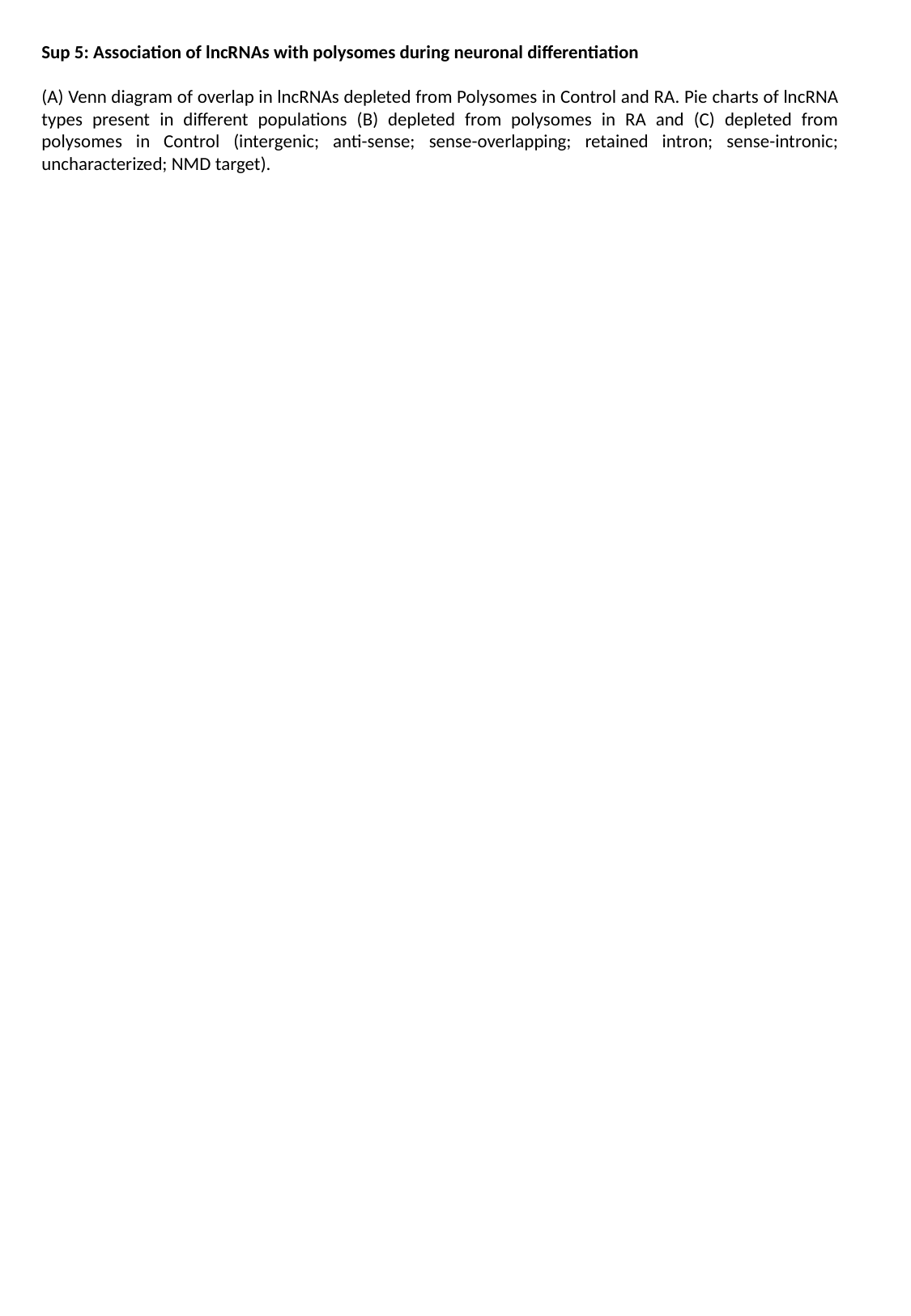

Sup 5: Association of lncRNAs with polysomes during neuronal differentiation
(A) Venn diagram of overlap in lncRNAs depleted from Polysomes in Control and RA. Pie charts of lncRNA types present in different populations (B) depleted from polysomes in RA and (C) depleted from polysomes in Control (intergenic; anti-sense; sense-overlapping; retained intron; sense-intronic; uncharacterized; NMD target).

## Slide 11
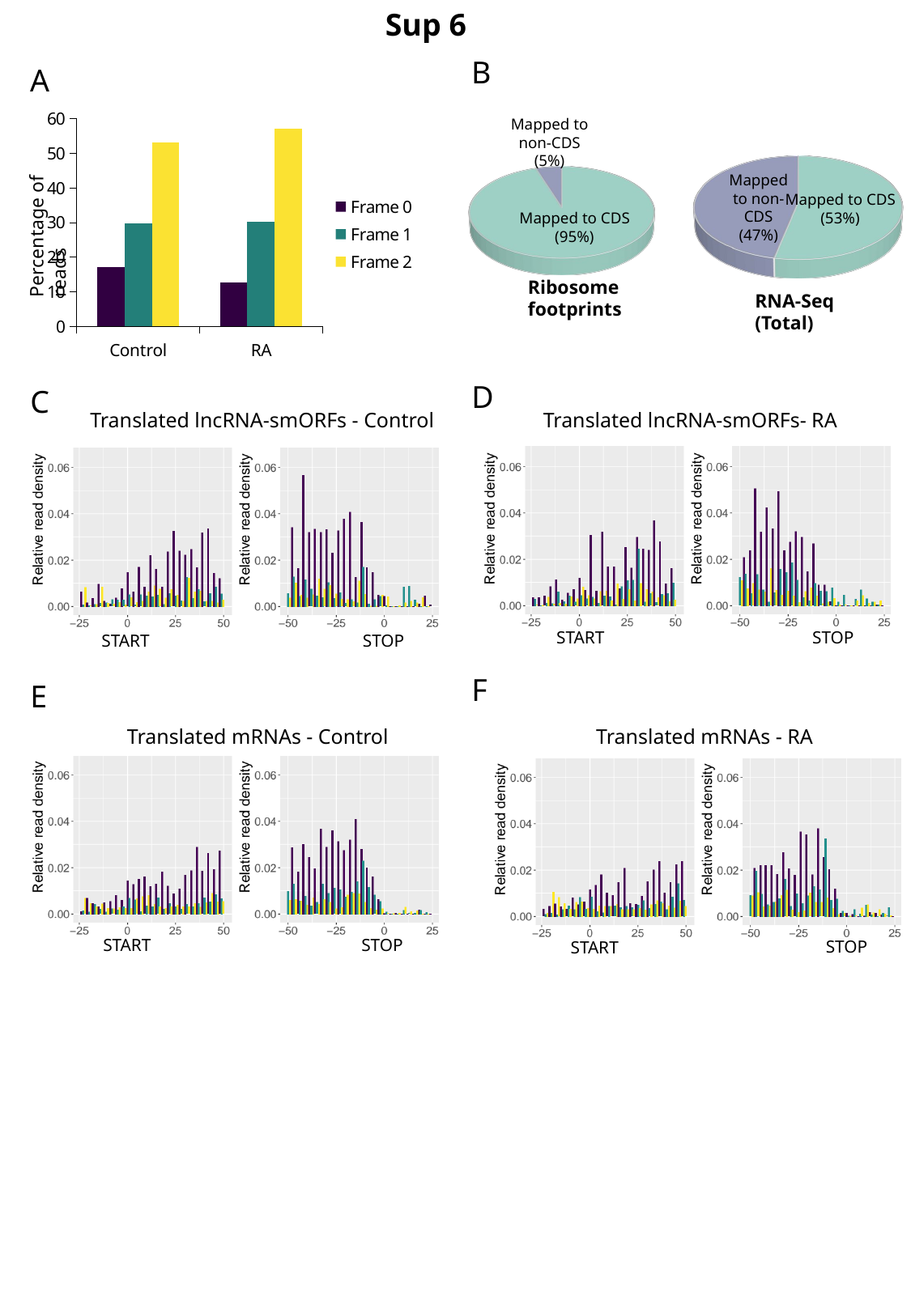

Sup 6
B
A
### Chart
| Category | Frame 0 | Frame 1 | Frame 2 |
|---|---|---|---|
| Control | 17.084737548310674 | 29.749887406578363 | 53.16537504511096 |
| RA | 12.708281117049516 | 30.248321042306664 | 57.04339784064382 |Mapped to non-CDS
(5%)
[unsupported chart]
[unsupported chart]
Ribosome footprints
RNA-Seq (Total)
Mapped to non-CDS
(47%)
Mapped to CDS
(53%)
Mapped to CDS
(95%)
Percentage of reads
D
C
Translated lncRNA-smORFs - Control
Translated lncRNA-smORFs- RA
STOP
START
START
STOP
F
E
Translated mRNAs - Control
Translated mRNAs - RA
START
STOP
STOP
START

## Slide 12
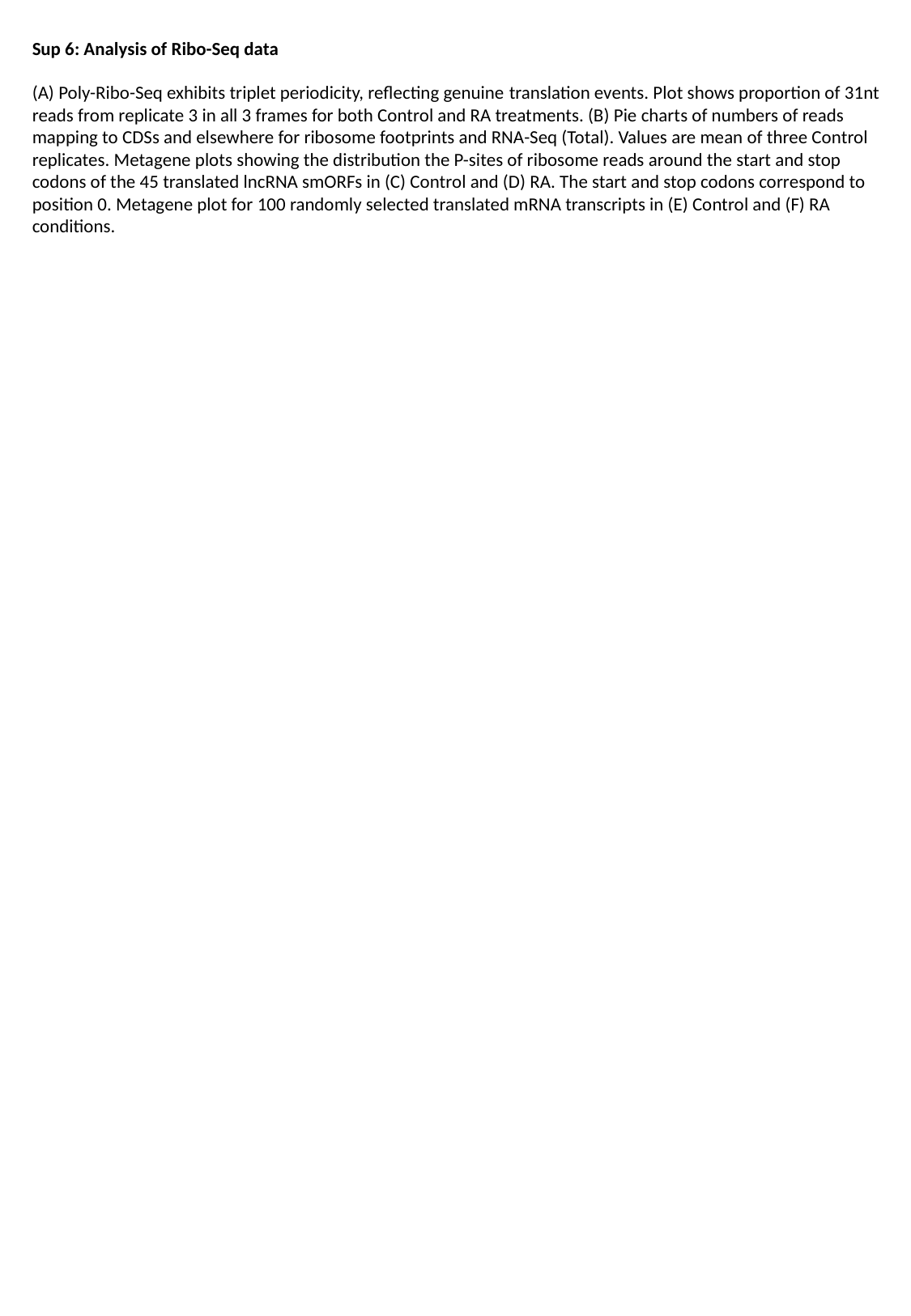

Sup 6: Analysis of Ribo-Seq data
(A) Poly-Ribo-Seq exhibits triplet periodicity, reflecting genuine translation events. Plot shows proportion of 31nt reads from replicate 3 in all 3 frames for both Control and RA treatments. (B) Pie charts of numbers of reads mapping to CDSs and elsewhere for ribosome footprints and RNA-Seq (Total). Values are mean of three Control replicates. Metagene plots showing the distribution the P-sites of ribosome reads around the start and stop codons of the 45 translated lncRNA smORFs in (C) Control and (D) RA. The start and stop codons correspond to position 0. Metagene plot for 100 randomly selected translated mRNA transcripts in (E) Control and (F) RA conditions.

## Slide 13
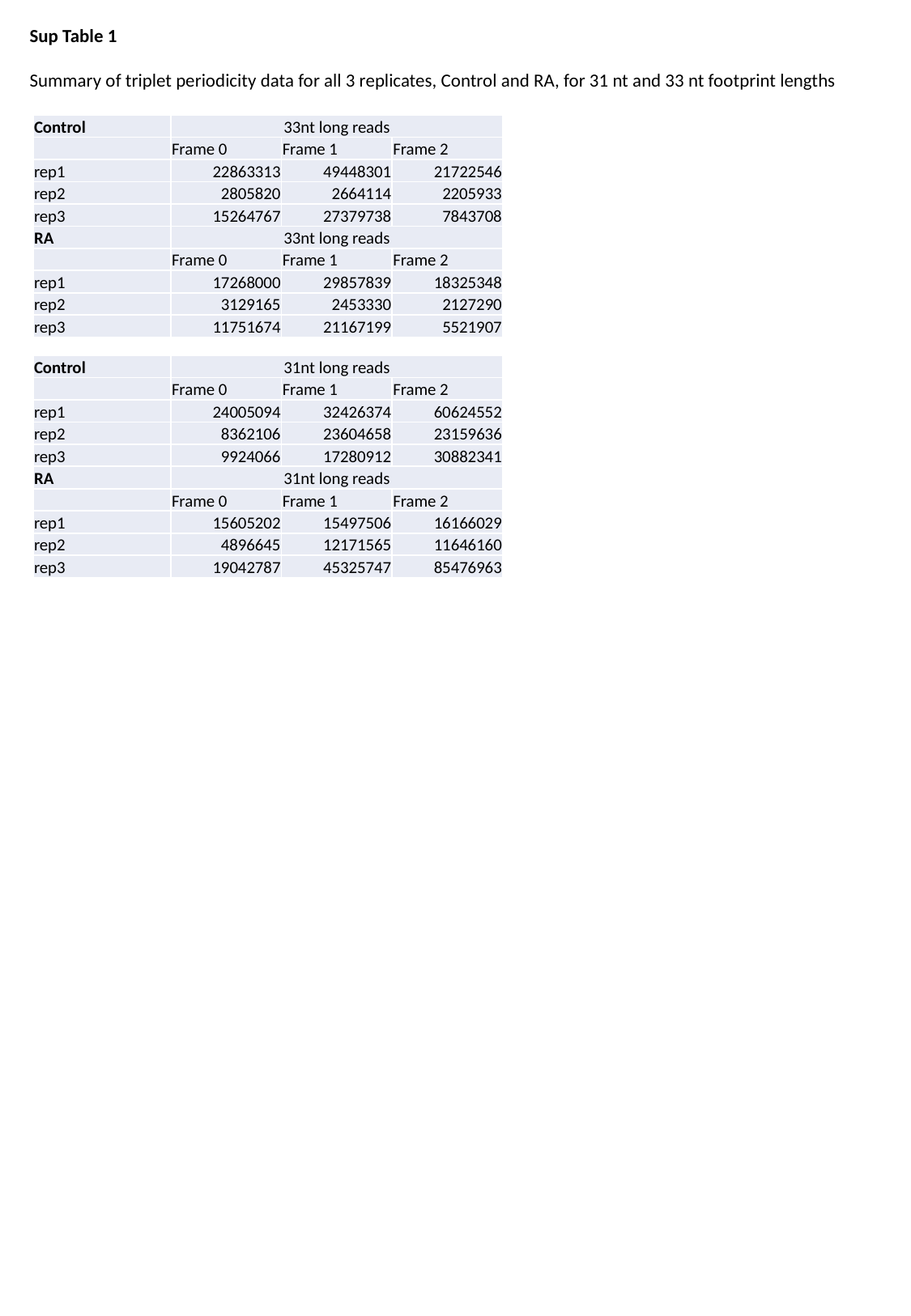

Sup Table 1
Summary of triplet periodicity data for all 3 replicates, Control and RA, for 31 nt and 33 nt footprint lengths
| Control | 33nt long reads | | |
| --- | --- | --- | --- |
| | Frame 0 | Frame 1 | Frame 2 |
| rep1 | 22863313 | 49448301 | 21722546 |
| rep2 | 2805820 | 2664114 | 2205933 |
| rep3 | 15264767 | 27379738 | 7843708 |
| RA | 33nt long reads | | |
| | Frame 0 | Frame 1 | Frame 2 |
| rep1 | 17268000 | 29857839 | 18325348 |
| rep2 | 3129165 | 2453330 | 2127290 |
| rep3 | 11751674 | 21167199 | 5521907 |
| Control | 31nt long reads | | |
| --- | --- | --- | --- |
| | Frame 0 | Frame 1 | Frame 2 |
| rep1 | 24005094 | 32426374 | 60624552 |
| rep2 | 8362106 | 23604658 | 23159636 |
| rep3 | 9924066 | 17280912 | 30882341 |
| RA | 31nt long reads | | |
| | Frame 0 | Frame 1 | Frame 2 |
| rep1 | 15605202 | 15497506 | 16166029 |
| rep2 | 4896645 | 12171565 | 11646160 |
| rep3 | 19042787 | 45325747 | 85476963 |

## Slide 14
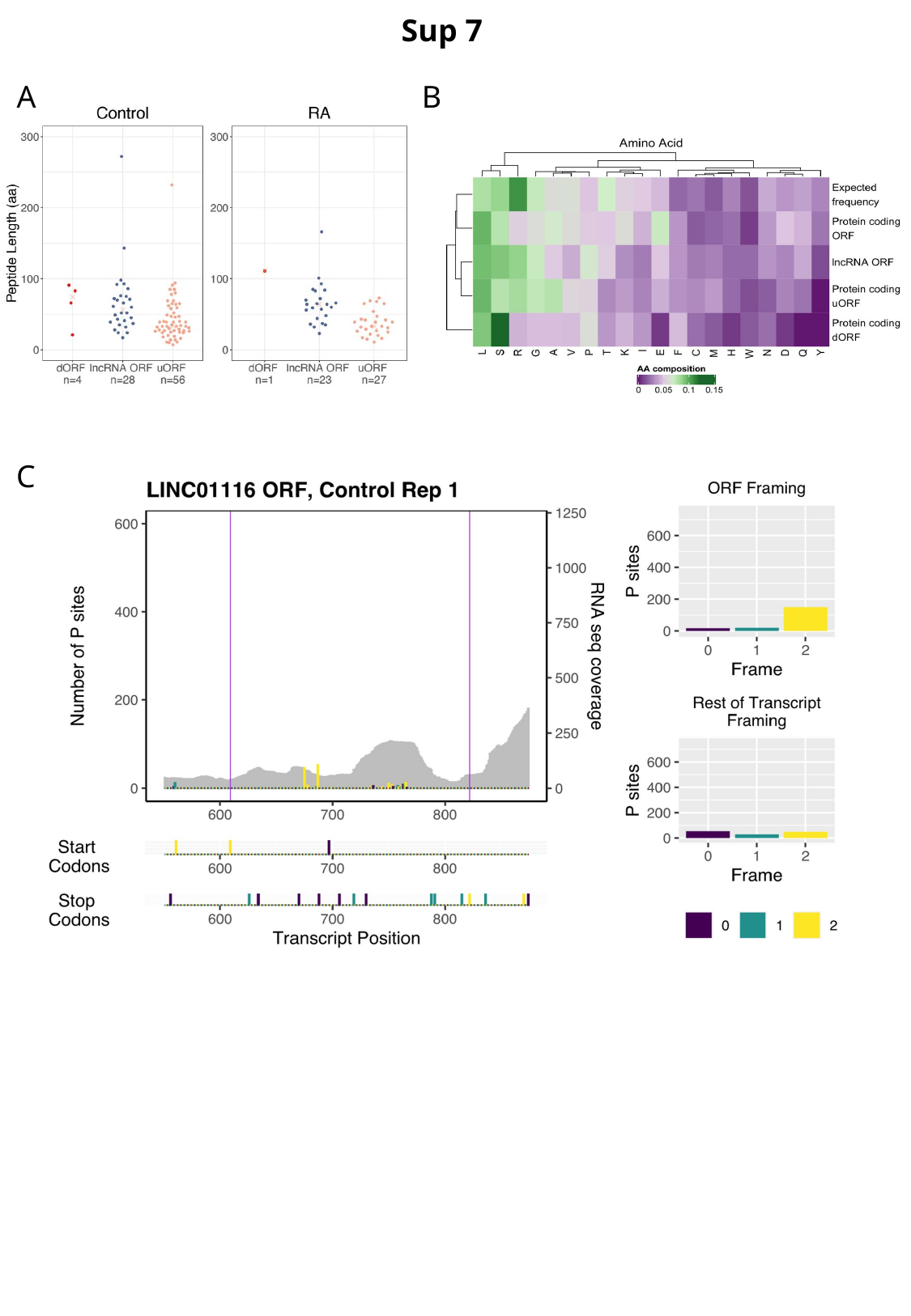

Sup 7
A
B
C

## Slide 15
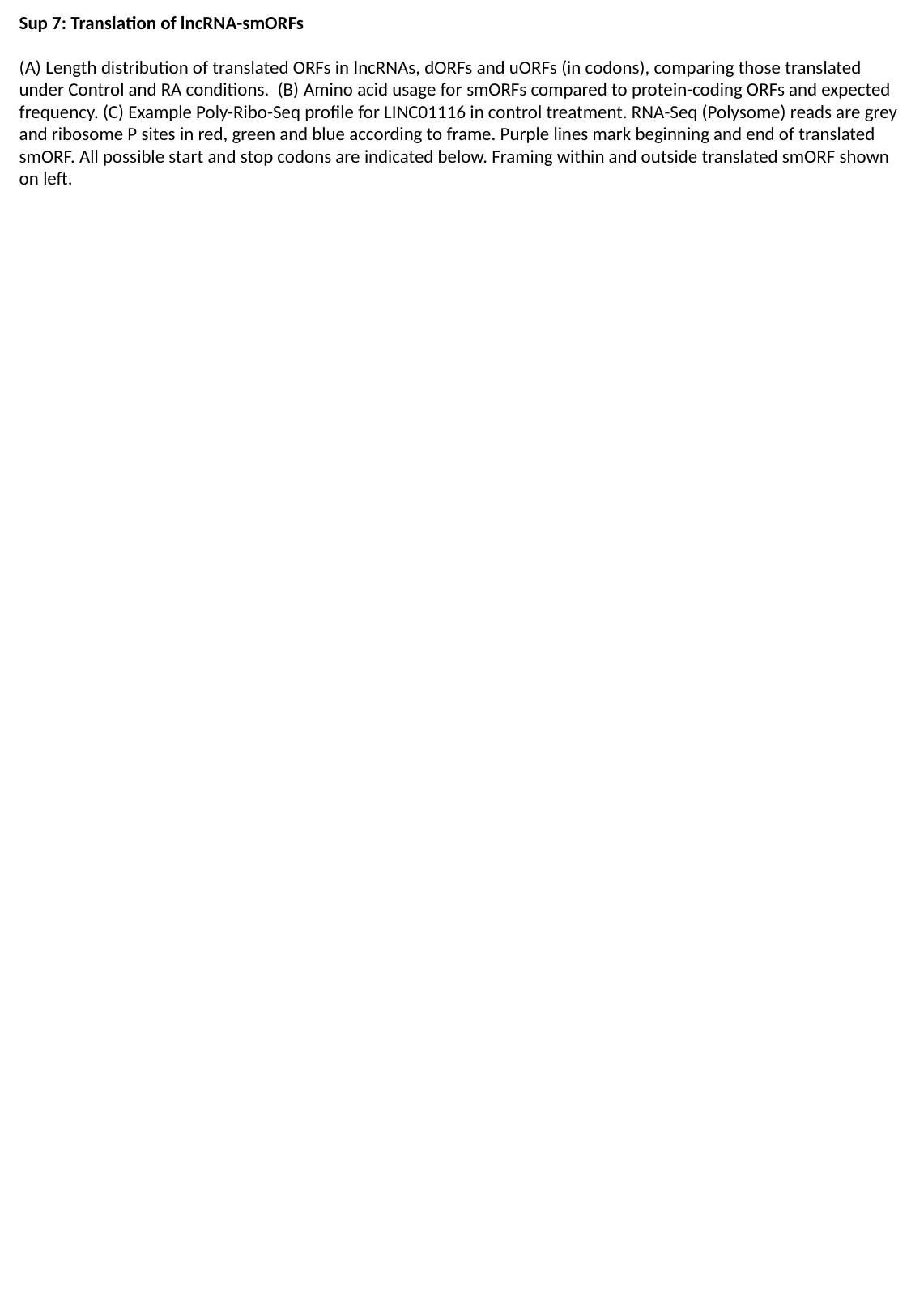

Sup 7: Translation of lncRNA-smORFs
(A) Length distribution of translated ORFs in lncRNAs, dORFs and uORFs (in codons), comparing those translated under Control and RA conditions. (B) Amino acid usage for smORFs compared to protein-coding ORFs and expected frequency. (C) Example Poly-Ribo-Seq profile for LINC01116 in control treatment. RNA-Seq (Polysome) reads are grey and ribosome P sites in red, green and blue according to frame. Purple lines mark beginning and end of translated smORF. All possible start and stop codons are indicated below. Framing within and outside translated smORF shown on left.

## Slide 16
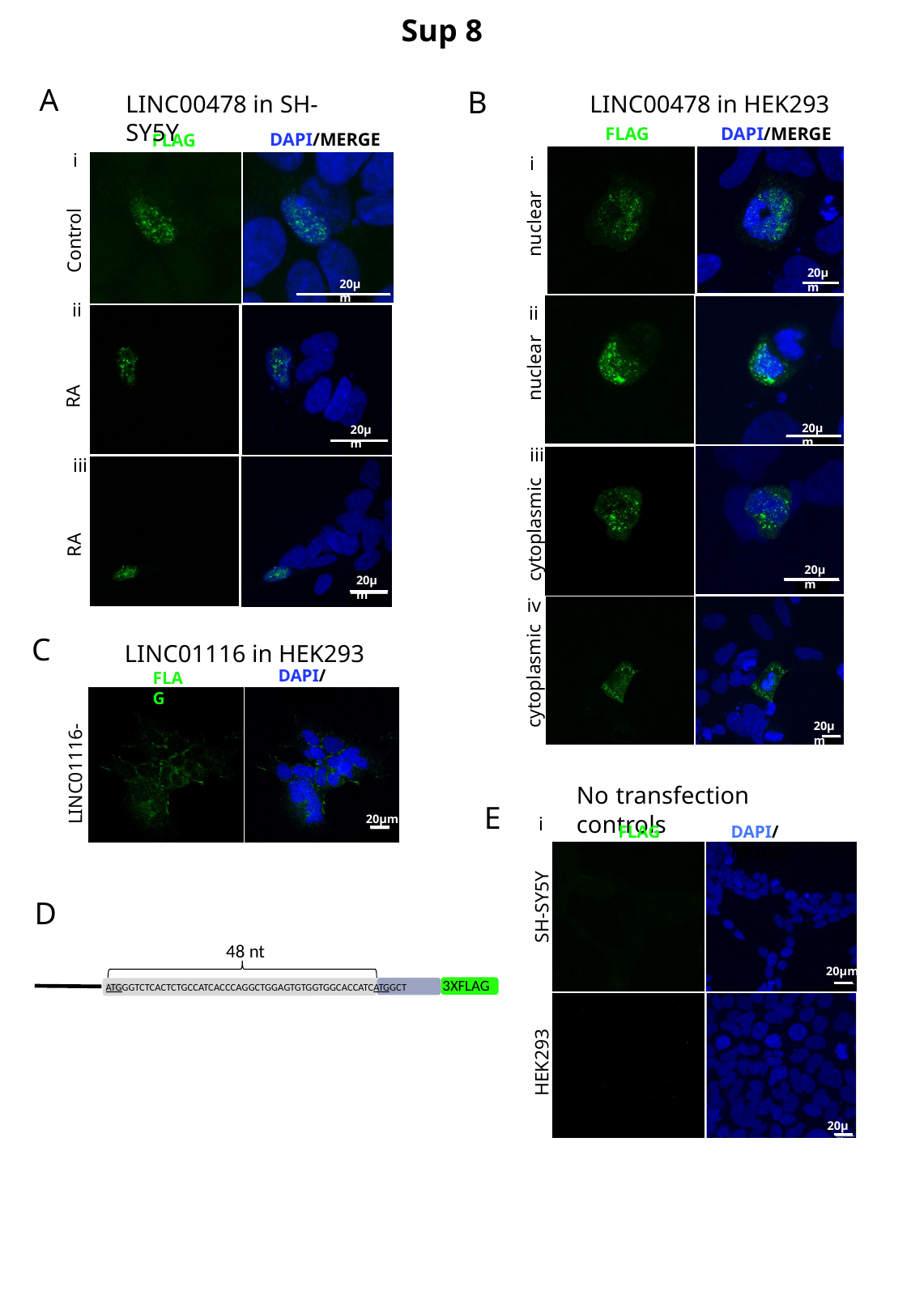

Sup 8
A
B
LINC00478 in HEK293
DAPI/MERGE
i
FLAG
nuclear
ii
nuclear
iii
cytoplasmic
20μm
20μm
20μm
iv
cytoplasmic
20μm
LINC00478 in SH-SY5Y
FLAG
i
DAPI/MERGE
Control
ii
RA
iii
RA
20μm
20μm
20μm
FLAG
DAPI/MERGE
C
LINC01116 in HEK293
DAPI/MERGE
FLAG
LINC01116-WT
20μm
No transfection controls
E
FLAG
DAPI/MERGE
SH-SY5Y
HEK293
20μm
20μm
i
D
48 nt
3XFLAG
ATGGGTCTCACTCTGCCATCACCCAGGCTGGAGTGTGGTGGCACCATCATGGCT
ii

## Slide 17
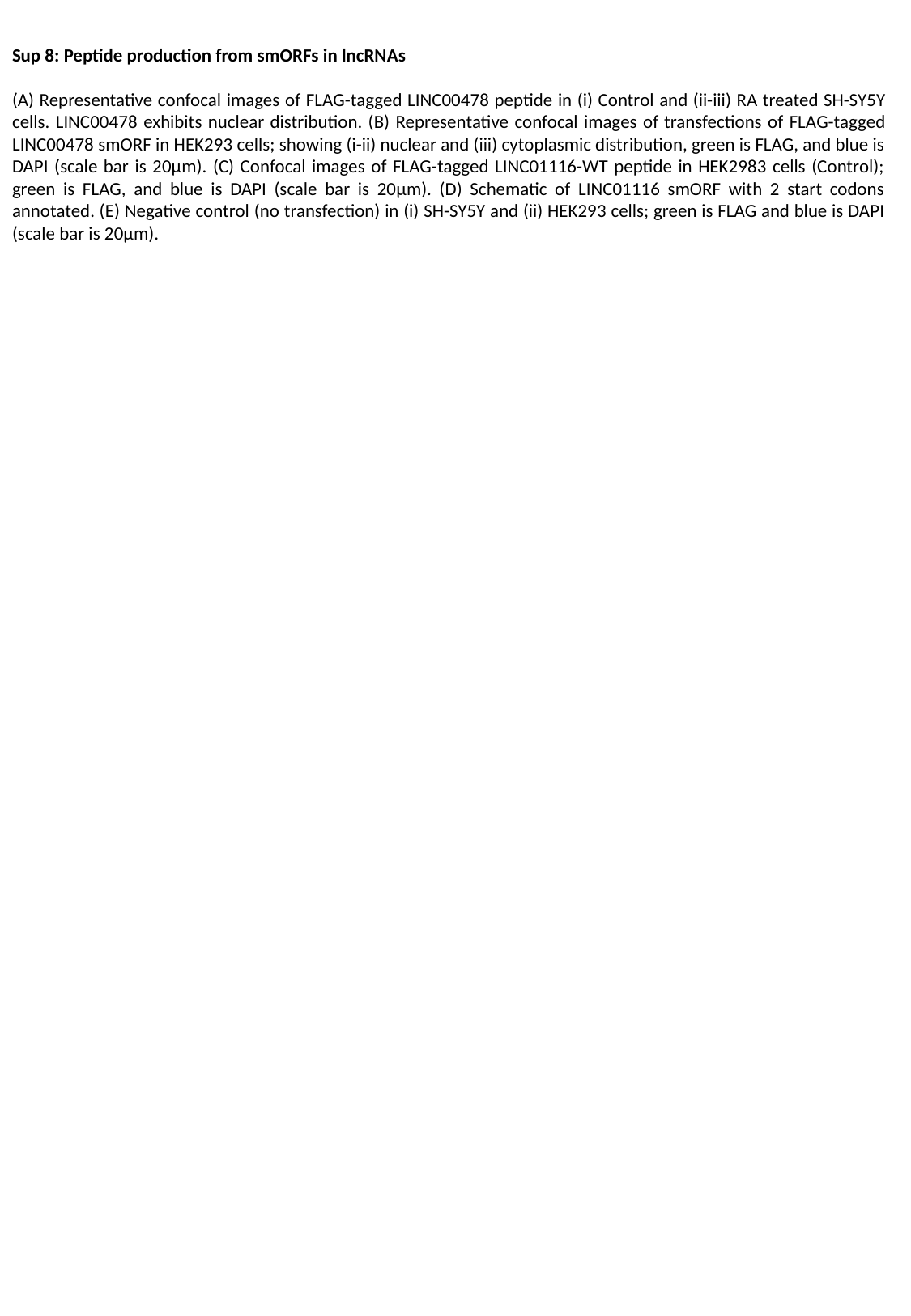

Sup 8: Peptide production from smORFs in lncRNAs
(A) Representative confocal images of FLAG-tagged LINC00478 peptide in (i) Control and (ii-iii) RA treated SH-SY5Y cells. LINC00478 exhibits nuclear distribution. (B) Representative confocal images of transfections of FLAG-tagged LINC00478 smORF in HEK293 cells; showing (i-ii) nuclear and (iii) cytoplasmic distribution, green is FLAG, and blue is DAPI (scale bar is 20μm). (C) Confocal images of FLAG-tagged LINC01116-WT peptide in HEK2983 cells (Control); green is FLAG, and blue is DAPI (scale bar is 20μm). (D) Schematic of LINC01116 smORF with 2 start codons annotated. (E) Negative control (no transfection) in (i) SH-SY5Y and (ii) HEK293 cells; green is FLAG and blue is DAPI (scale bar is 20μm).

## Slide 18
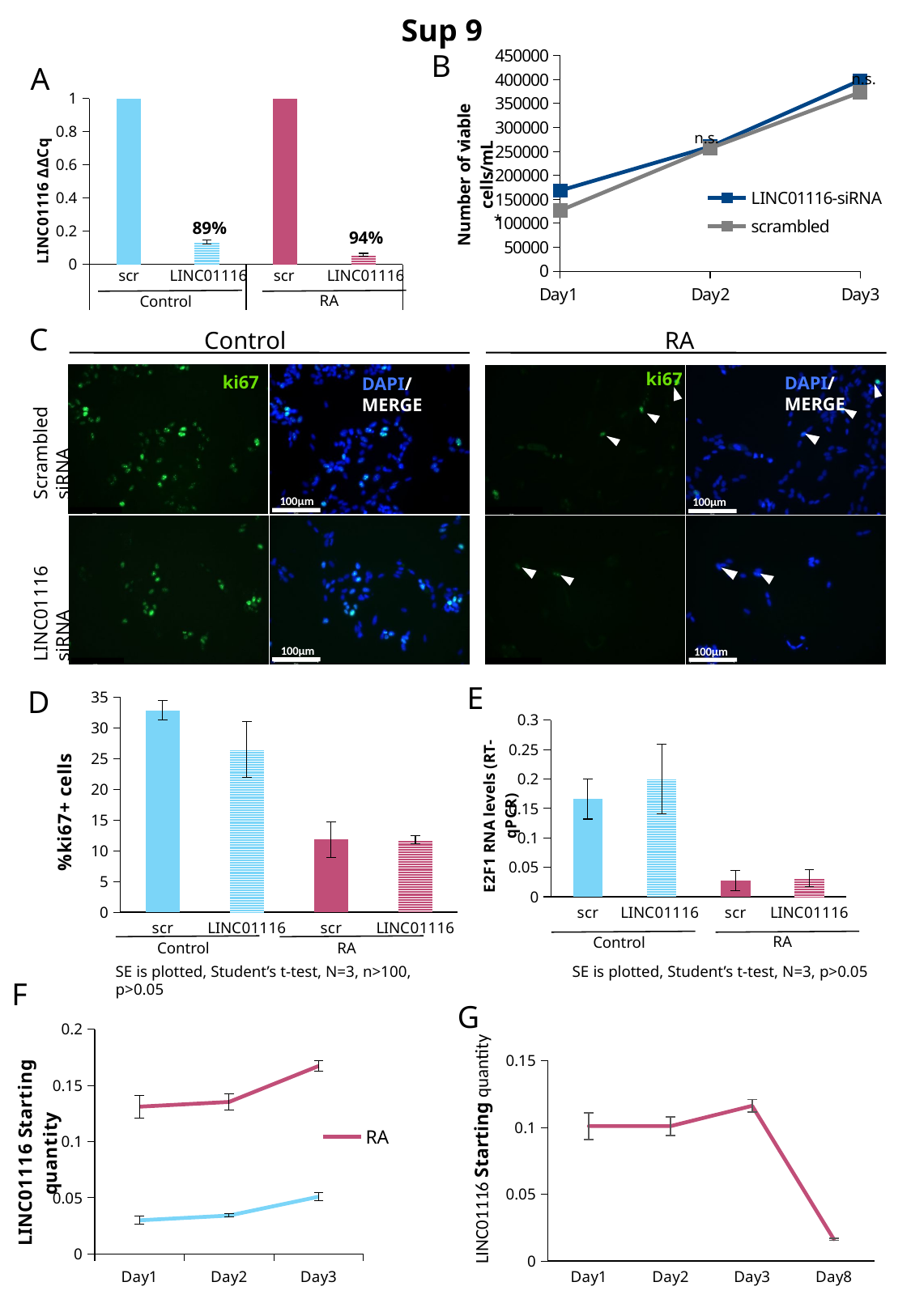

Sup 9
### Chart
| Category | LINC01116-siRNA | scrambled |
|---|---|---|
| Day1 | 168333.333333333 | 126666.666666667 |
| Day2 | 260000.0 | 256666.666666667 |
| Day3 | 398333.333333333 | 373333.333333333 |n.s.
n.s.
*
B
A
### Chart
| Category | ΔΔCq expression |
|---|---|
| scrambled siRNA | 1.0 |
| LINC01116 siRNA | 0.135857658098091 |
| scrambled siRNA | 1.0 |
| LINC01116 siRNA | 0.0581782396078611 |89%
94%
scr
LINC01116
scr
LINC01116
RA
Control
C
Control
RA
ki67
ki67
DAPI/MERGE
DAPI/MERGE
Scrambled siRNA
LINC01116 siRNA
100μm
100μm
100μm
100μm
E
### Chart
| Category | E2F1 |
|---|---|
| scr | 0.16590261370513 |
| LINC01116 | 0.200380322989864 |
| scr | 0.0274731928236441 |
| LINC01116 | 0.0321057987953723 |RA
Control
SE is plotted, Student’s t-test, N=3, p>0.05
D
### Chart
| Category | %ki67+ cells |
|---|---|
| scr | 32.88084128553783 |
| LINC01116 | 26.4569616375829 |
| scr | 11.8446159730108 |
| LINC01116 | 11.8133254908094 |RA
Control
SE is plotted, Student’s t-test, N=3, n>100,  p>0.05
F
G
### Chart
| Category | Control | RA |
|---|---|---|
| Day1 | 0.0299302657001471 | 0.10119990538965 |
| Day2 | 0.0341819443789818 | 0.101165923211835 |
| Day3 | 0.0509417692126467 | 0.116412145843012 |
### Chart
| Category | |
|---|---|
| Day1 | 0.10119990538965 |
| Day2 | 0.101165923211835 |
| Day3 | 0.116412145843012 |
| Day8 | 0.0163557657812928 |LINC01116 Starting quantity

## Slide 19
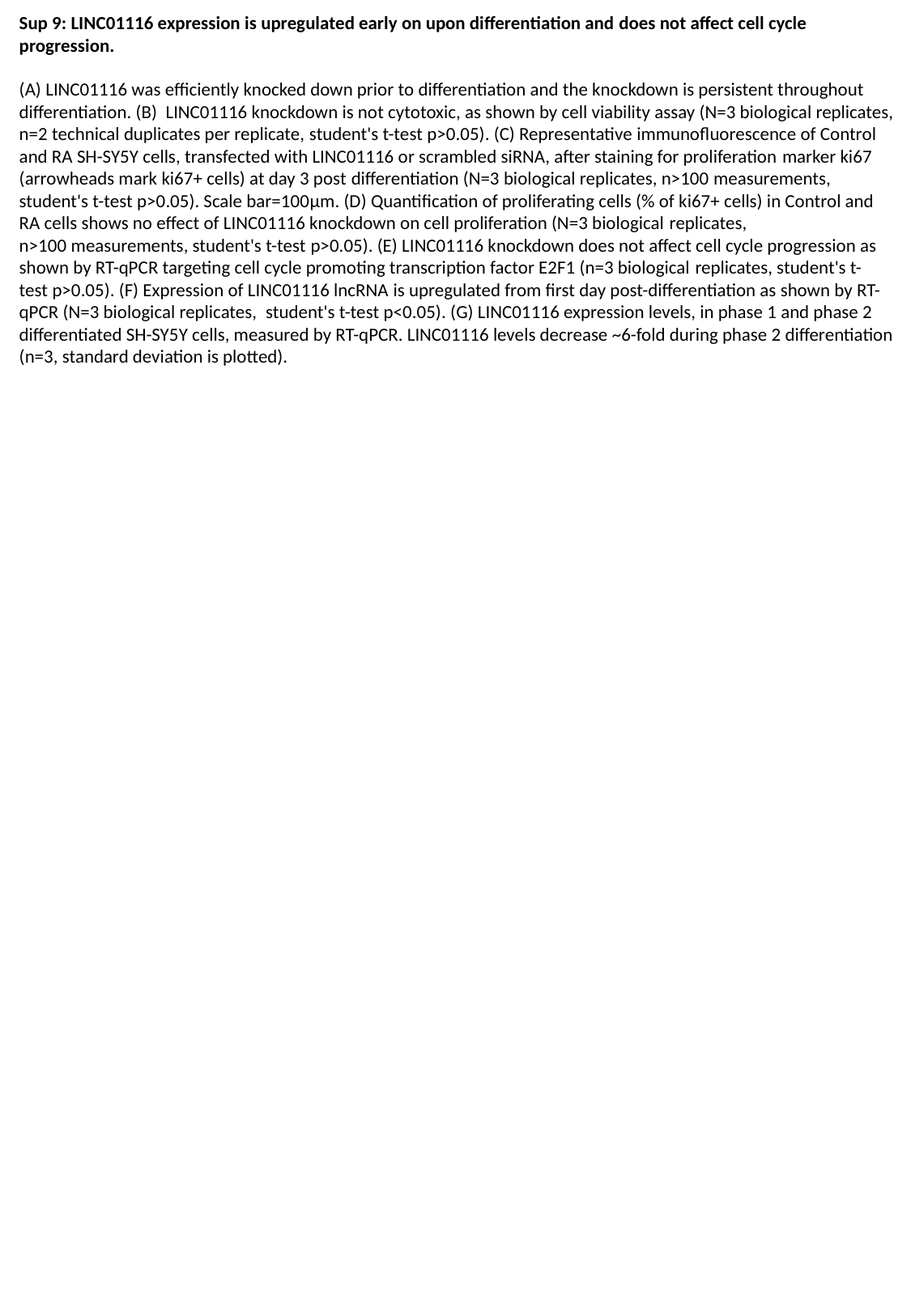

Sup 9: LINC01116 expression is upregulated early on upon differentiation and does not affect cell cycle progression.
(A) LINC01116 was efficiently knocked down prior to differentiation and the knockdown is persistent throughout differentiation. (B) LINC01116 knockdown is not cytotoxic, as shown by cell viability assay (N=3 biological replicates, n=2 technical duplicates per replicate, student's t-test p>0.05). (C) Representative immunofluorescence of Control and RA SH-SY5Y cells, transfected with LINC01116 or scrambled siRNA, after staining for proliferation marker ki67 (arrowheads mark ki67+ cells) at day 3 post differentiation (N=3 biological replicates, n>100 measurements, student's t-test p>0.05). Scale bar=100μm. (D) Quantification of proliferating cells (% of ki67+ cells) in Control and RA cells shows no effect of LINC01116 knockdown on cell proliferation (N=3 biological replicates, n>100 measurements, student's t-test p>0.05). (E) LINC01116 knockdown does not affect cell cycle progression as shown by RT-qPCR targeting cell cycle promoting transcription factor E2F1 (n=3 biological replicates, student's t-test p>0.05). (F) Expression of LINC01116 lncRNA is upregulated from first day post-differentiation as shown by RT-qPCR (N=3 biological replicates,  student's t-test p<0.05). (G) LINC01116 expression levels, in phase 1 and phase 2 differentiated SH-SY5Y cells, measured by RT-qPCR. LINC01116 levels decrease ~6-fold during phase 2 differentiation (n=3, standard deviation is plotted).
