# Supplementary material for: Cytoplasmic long noncoding RNAs are differentially regulated and translated during human neuronal differentiation
Source: RNA. 2021 Sep;27(9):1082–101. doi: 10.1261/rna.078782.121 (PMC8370745; doi:10.1261/rna.078782.121)
Supplement: Supplemental Material [file supp_078782.121_Supplemental_Methods.docx]

**Supplementary methods**

Antibody Table

| **Protein** | **Species** | **Company** | **Usage (ICC/WB)** | **Dilution** |
| --- | --- | --- | --- | --- |
| betaIII-tubulin (TuJ1) | rabbit | ProteinTech | ICC | 1:50 |
| ki67 | mouse | Dako-Agilent | ICC | 1:100 |
| c-Fos | rabbit | Santa Cruz | ICC | 1:50 |
| Tubulin B | mouse | DSHB | both | 1:5000 for WB, 1:200 for ICC |
| NXF1 | mouse | abcam | WB | 1:5000 |
| H3K27me3 | mouse | abcam | WB | 1:1000 |
| FLAG | mouse | Sigma | both | 1:1000 |
| hnRNPK | rabbit | abcam | ICC | 1:100 |
| anti-mouse IgG-HRP | goat | New England Biolabs | WB | 1:5000 |
| anti-rabbit IgG-HRP | goat | New England Biolabs | WB | 1:5000 |
| Alexa anti-mouse IgG-488 | goat | Thermofisher | ICC | 1:500 |
| Alexa anti-rabbit IgG-488 | goat | Thermofisher | ICC | 1:500 |
| Alexa anti-rabbit 633 | goat | Thermofisher | ICC | 1:500 |

qPCR primer Table

| **qPCR** |  |  |
| --- | --- | --- |
| gene/transcript | Forward primer sequence | Reverse primer sequence |
| MOXD1 | GGAAGCCGAAAAGCCAAGTG | TCGAAAATGACGCAGCCTGA |
| NTN4 | CGAGTGCAGAACCTGCAAGTGT | CATCTGGAGCTGAGAAGGGTC |
| E2F1 | TGGAGCAAGAACCGCTGTTGT | GGGAAAGGCTGATGAACTCCT |
| SOX2 | ACATGAACGGCTGGAGCAA | GTAGGACATGCTGTAGGTGGG |
| GAPDH | CATCCTGGGCTACACTGAGC | GTCAAAGGTGGAGGAGTGGG |
| LINC01116 | TCTAAGAATGGGTCTCACTCTGC | CCAGGCATGGTGGCTCAC |
| LINC02143 | AACCTTTGCAGTAGCTCCTGG | GGATGAGGAGACTGAGACTGAGAG |
| AC254633.1 | GTGACTCACCTCCCAGACTTC | TGCTGTGCAGCCAGCGTC |
| DLGAP1-AS1 | TCTGAGAGCCAGCGAACTTT | AGCCTGTTGCGTCATGTGAT |
| DLGAP1-AS2 | CCCAGGACACAGACAAGACC | ATGCACGCTCTCTGACAGCA |
| SERPINB9P1 | AGTCAGCGAGTGGACAAAGC | GACTCCATGCTGCGGTTTTC |
| AC090001.1 | GTGCCCATGAGGGAGAACAC | GACAAGAAGTCAGGAGGTAGACA |
| SNAP25-AS1 | AGCCATGGAAGTCAAATGCTG | AGGCATTTTTGCTGTCTTTCCTC |
| XIST | GGCTCCTCTTGGACATTCTGAG | AGCTTGGCCAGATTCTCAAAG |

Buffer Table

| **Polysome lysis buffer (**1mL/70 million cells) |  |
| --- | --- |
| Component | final concentration |
| Tris-HCl pH8 | 50mM |
| NaCl | 150mM |
| MgCl_2_ | 10mM |
| DTT | 1mM |
| IGEPAL | 1% |
| cycloheximide | 100µg/mL |
| Turbo DNase | 24U/mL |
| RNase Inhibitor (RNaseInPlus) | 90U |
| cOmplete Protease Inhibitor (Roche) | 0.33% |
| ddH2O |  |
|  |  |
| **RNaseI footprinting buffer** |  |
| Component | final concentration |
| Tris-HCl pH8 | 100mM |
| NaCl | 30mM |
| MgCl_2_ | 10mM |
| RNaseI (EN0601-Thermo) | 0.8-1U/million cells |
|  |  |
| SuperRNase Inhibitor (Ambion) | 3U/million cells |
|  |  |
| **Cytoplasmic/Nuclear fractionation buffer** |  |
| Component | final concentration |
| Phosphate Buffer Saline (PBS) | 1X |
| Triton-X | 1% |
| RNase Inhibitor (RNaseInPlus) | 40U/mL |
| ddH2O |  |
|  |  |
| **Nuclei lysis buffer-RIPA based** |  |
| Component | final concentration |
| NaCl | 150mM |
| IGEPAL | 1% |
| Sodium Deoxycholate (DOC) | 0.5% |
| SDS | 0.1% |
| Tris-HCl pH 7.4 | 25mM |
| cOmplete Protease Inhibitor (Roche) | 1X |
| ddH2O |  |

Cloning Primer Table

| Transcript | Forward primer sequence | Reverse primer sequence |
| --- | --- | --- |
| LINC001116-smORF | GGCGGTGCTAGCGCGAGCCACGGGCCTC | GGCGGTGATATCTCACTTGTCATCGTCATCCTTGTAATCGATGTCATGATCTTTATAATCACCGTCATGGTCTTTGTAGTCGTTTTTAAGCTGACTTGTC |
| LINC01116-whole | GGCGGTGCTAGCGCGAGCCACGGGCCTC | ACCGCCGATATCCAATTACTGCATTCACGTATTCTTC |
| LINC00478 | GGCGGTGCTAGCCTCCTGTCGTTAAGATAAATTCTCCA | GGCGGTGATATCTCACTTGTCATCGTCATCCTTGTAATCGATGTCATGATCTTTATAATCACCGTCATGGTCTTTGTAGTCGTAGTAAAATGCTCTCTGA |
| LINC01116- mutant (ATG1) | CTTCTAAGAAAAGGTCTCACTCTGC | CAATTCAGTTGTCTTCTAATAC |
| LINC01116- mutant (ATG2) | GGCACCATCAAAGCTCACTGCAGC | ACCACACTCCAGCCTGGG |
| LINC01116-mutant (both ATG) | GCTGGAGTGTGGTGGCACCATCAAAGCTCACTGCAGCCTTGAA | CTGGGTGATGGCAGAGTGAGACCTTTTCTTAGAAGCAATTCAGTTGTC |
